# Attention-based deep clustering method for scRNA-seq cell type identification

**DOI:** 10.1371/journal.pcbi.1011641

**Published:** 2023-11-10

**Authors:** Shenghao Li, Hui Guo, Simai Zhang, Yizhou Li, Menglong Li

**Affiliations:** 1 College of Chemistry, Sichuan University, Chengdu, Sichuan, China; 2 West China Biomedical Big Data Center, West China Hospital, Sichuan University, Sichuan, China; 3 School of Cyber Science and Engineering, Sichuan University, Chengdu, Sichuan, China; University of Washington, UNITED STATES

## Abstract

Single-cell sequencing (scRNA-seq) technology provides higher resolution of cellular differences than bulk RNA sequencing and reveals the heterogeneity in biological research. The analysis of scRNA-seq datasets is premised on the subpopulation assignment. When an appropriate reference is not available, such as specific marker genes and single-cell reference atlas, unsupervised clustering approaches become the predominant option. However, the inherent sparsity and high-dimensionality of scRNA-seq datasets pose specific analytical challenges to traditional clustering methods. Therefore, a various deep learning-based methods have been proposed to address these challenges. As each method improves partially, a comprehensive method needs to be proposed. In this article, we propose a novel scRNA-seq data clustering method named AttentionAE-sc (Attention fusion AutoEncoder for single-cell). Two different scRNA-seq clustering strategies are combined through an attention mechanism, that include zero-inflated negative binomial (ZINB)-based methods dealing with the impact of dropout events and graph autoencoder (GAE)-based methods relying on information from neighbors to guide the dimension reduction. Based on an iterative fusion between denoising and topological embeddings, AttentionAE-sc can easily acquire clustering-friendly cell representations that similar cells are closer in the hidden embedding. Compared with several state-of-art baseline methods, AttentionAE-sc demonstrated excellent clustering performance on 16 real scRNA-seq datasets without the need to specify the number of groups. Additionally, AttentionAE-sc learned improved cell representations and exhibited enhanced stability and robustness. Furthermore, AttentionAE-sc achieved remarkable identification in a breast cancer single-cell atlas dataset and provided valuable insights into the heterogeneity among different cell subtypes.

## Author summary

Single-cell sequencing (scRNA-seq) has been widely used in numerous biological studies to reveal heterogeneity at the cellular level. Accurate cell type identification serves as the foundation for scRNA-seq data analysis, and unsupervised cluster analysis is commonly employed when an appropriate reference is not available. However, the inherent sparsity and high-dimensionality of scRNA-seq datasets pose specific analytical challenges to traditional clustering methods. To address this, we propose a novel scRNA-seq data clustering method named AttentionAE-sc (Attention fusion AutoEncoder for single-cell). By integrating denoising representation learning and cluster-friendly representation learning through an attention mechanism, AttentionAE-sc demonstrated outstanding performance in the evaluation phase. Firstly, when compared to benchmark methods on the real scRNA-seq datasets, AttentionAE-sc consistently achieved superior external and internal clustering evaluation metrics. Secondly, AttentionAE-sc exhibited robustness and stability across various experimental conditions. Lastly, AttentionAE-sc not only delivered excellent clustering results but also unveiled potential biological insights on a breast cancer single-cell atlas dataset.

## 1. Introduction

Single Cell RNA sequencing (scRNA-seq) techniques facilitate exploring the heterogeneity and diversity of cells at a cellular level, providing a more detailed mechanism for a variety of biological researches [1, 2]. Accurate identification of cell subpopulation is essential for many downstream analyses and several auxiliary tools have been constructed [3, 4]. The basic cell subpopulations apportion capability of these tools is based on unsupervised clustering, which is a feasible solution for scRNA-seq datasets lack of cell atlas [5]. However, major computational challenges in scRNA-seq analysis include the high sparsity, high dimensionality and batch effects [6, 7]. As a result, more and more clustering methods have been developed for cell group assignments and jointly dealing with problems mentioned above [8, 9, 10, 11], but an optimal approach has not yet been proposed.

In the last few years, various clustering methods involved in other fields have been applied to scRNA-seq datasets. For example, RaceID [12] applied K-means to the clustering analysis of scRNA-seq datasets to groups identification and introduces the outlier detection to improve the ability of K-means for race cells recognition. However, K-means inevitably converges to the local minimization because the process of searching cluster center is sensitive to initialization. SC3 [13] overcame the problems by integrating multiple results after running K-means repeatedly. Another type of method supported by various scRNA-seq data analysis tools [4] is based on community detection, such as Louvain or Leiden algorithm [14, 15], which iteratively aggregates the nodes in the network composed of cells into multiple communities and gradually gets the categories of all cells. The major advantage of these algorithms based on community discovery over other methods is that they don’t need the information on the number of groups, which makes them more suitable for the scRNA-seq dataset with scarce prior knowledge.

To efficiently integrate single-cell clustering methods with dimension reduction, data denoising and batch effects removal processes, various deep learning-based tools have been proposed. DCA [16] raised a denoising autoencoder (DAE) to denoise and impute the original scRNA-seq data by minimizing a zero-inflated negative binomial (ZINB) distribution loss. Additionally, scDeepCluster [9] combined deep embedding clustering (DEC) [17] to concurrently learn feature representation and optimize clustering performance. DEC is a self-optimizing clustering method and can provide the soft labels of cells. Moreover, DESC [18] was composed of stacked autoencoders and DEC. They discussed the biological interpretable soft assignment performed by DEC and further analyzed the effectiveness of soft clustering on batch effects removal. ScVI (Single-cell Variational Inference) [19] approximated the ZINB distribution of expression values and a comprehensive tool called scvi-tools [20] was proposed to integrate multiple methods for various tasks in single-cell dataset analysis. However, the interaction among cells wasn’t directly employed in the feature extraction of each cell by these methods. To explicitly incorporate cell-to-cell information, graph neural network (GNN) has been employed in scRNA-seq clustering analysis [10, 21] to extract a topological embedding of cells, which captured relationship among cells and can significantly enhance the learning of clustering-friendly representations [11].

Different from the previous methods, GNN-based methods need to manually construct the topologic information between cells in the absence of prior knowledge. Two types of graphs are mainly included: cell-gene graph and cell-cell graph. scDeepSort [22] proposed a strategy to construct the cell-gene graph using gene expression values as edges, and a weighted GNN-based model was constructed for cell annotation. graph-sc [21] also used a similar component graph strategy and graph autoencoder (GAE) [23] to perform the clustering analysis of scRNA-seq data. Besides, the cell-cell graph was used in scGNN [10] and represented the cells as graph nodes only. The connection among cells was obtained by a multi-modal autoencoders in an iterative way. Same as graph-sc, scGNN extracted the output features of graph encoder, representation of cells in the low dimensional hidden space, and obtained the clustering results relying on the separate K-means or community detection algorithms. scGAC [11] also constructed the cell-cell graph and adopted a self-optimizing method to simultaneously learn representation and optimize clustering. The topology embeddings of cells were extracted by Graph Attention Network-based GAE, and the optimized clustering results were obtained by DEC. Additionally, SCEA utilized an Encoder based on Multilayer Perceptron (MLP) for data dimension reduction prior to employing a similar topology representation learning strategy and achieved superior performance [24].

**Fig 1:**
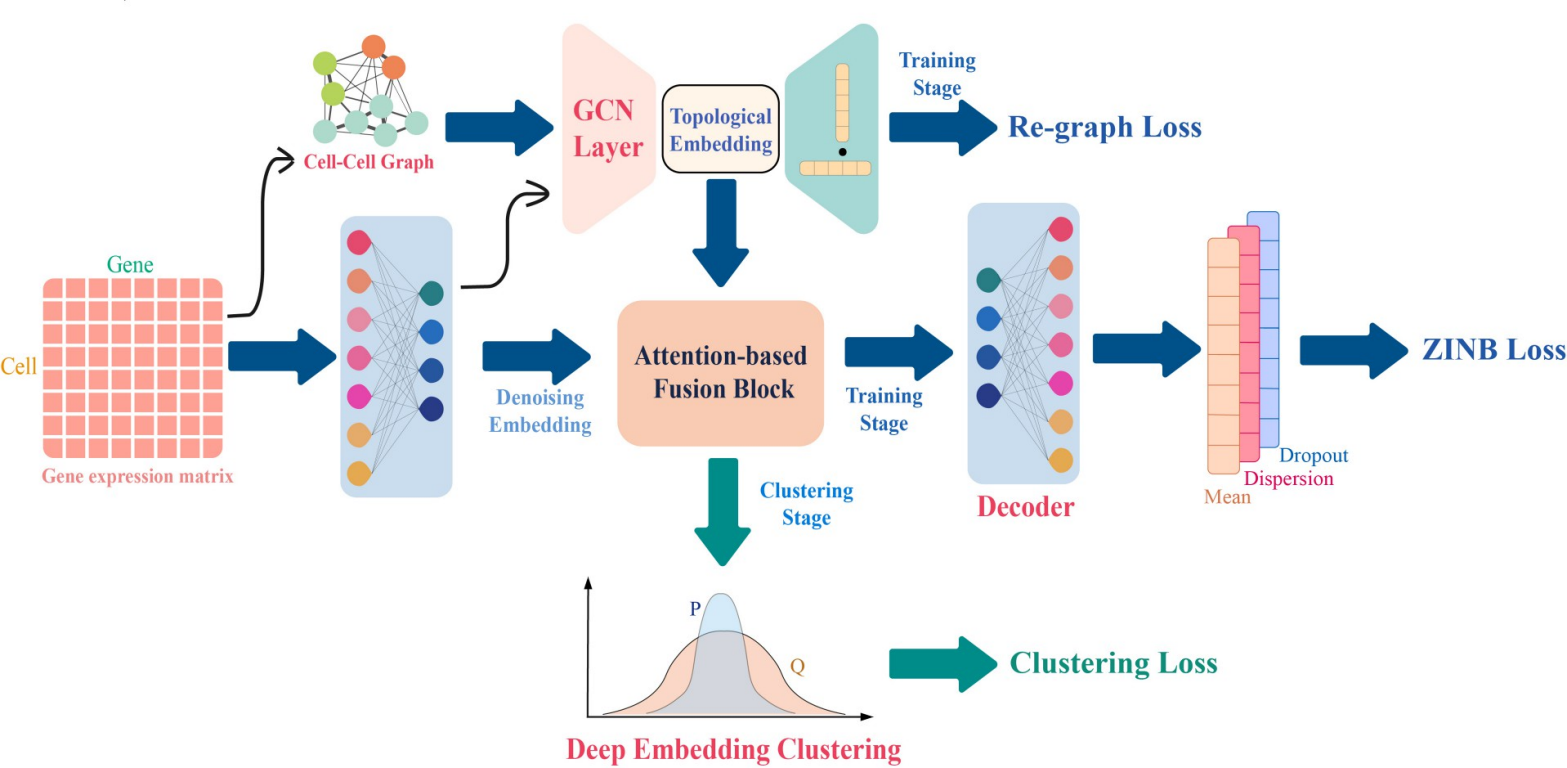
Model architecture. In the train stage, a graph autoencoder (GAE) and a denoising autoencoder (DAE) are constructed simultaneously. The multi-head attention blocks are used to combine the denoising embedding and the topological embedding. In the clustering stage, the prediction labels of each cell are obtained by a self-optimizing clustering.

Motivated by the researches above, we propose an attention mechanism-based scRNA-seq clustering method named AttentionAE-sc (Attention fusion AutoEncoder for single-cell). As shown in Fig 1, DAE and GAE are implemented simultaneously to learn the cellular embeddings. Direct use of topology embedding in the clustering phase is easily affected by noisy edges. Therefore, an information fusion block based on multi-head attention mechanism is suggested to combine the topological information and the denoising information to reconstruct the relationships among cells (S1 Fig). Through simultaneous learning different embeddings from scRNA-seq datasets, the information fusion blocks are iteratively optimized to learn the clustering-friendly representations so that similar cells are closer in the hidden embedding for better clustering analysis. To obtain a soft subpopulation partition, a self-optimizing clustering process is conducted through DEC. By initializing the cluster centers with Leiden algorithm, we can obtain the allocation labels of cells without specifying the number of groups. AttentionAE-sc achieved an excellent performance on 16 scRNA-seq datasets. Firstly, AttentionAE-sc completely outperformed other methods based on community detection algorithm in terms of clustering performance. To our surprise, it even achieved superior results when compared to several K-means-based methods that require specifying the number of groups in advance. Secondly, AttentionAE-sc was capable of learning a more clustering-friendly representation compared to other DEC-based methods (or methods without DEC), showcasing its robustness and stability when subjected to random perturbations such as down-sampling or manual dropout. Finally, we applied AttentionAE-sc to conduct clustering analysis on a large breast cancer single-cell atlas dataset, acquiring promising results in terms of the degree of similarity between the clustering outcomes and real cell types, as well as the biological significance captured by the predicted labels.

## 2. Materials & methods

### 2.1 Data preprocessing

The preprocessing process was implemented by *scanpy* package [4]. Given a raw count matrix ***R***_*N*×*M*_ (N cells and M genes), cells or genes without any counts are filtered (n cells leftover). After normalization for each cell, data is smoothed by simple scaling. Only the most variable genes (top 2500) were extracted as gene expression matrix ***X***_*n*×2500_, which was used for cell-cell graph construction. And its corresponding original count matrix ${R^{\prime }}_{n\times 2500}$ was used for DAE training. Finally, ***X***_*n*×*m*_ was transformed into z-score data ***X***_*z*-*score*_ so that the mean value of each selected gene was zero and the variance was the unit value. In addition, the adjacency matrix ***A***_*n*×*n*_, another input of AttentionAE-sc, was calculated by measuring the distance *d*_*ij*_ among cells (the distance between cell *i* and cell *j*) with the Gaussian kernel, which is a decreasing function with the distance *d*_*ij*_:

$$A_{ij}=\exp\left(-\frac{||d_{ij}|{|}^{2}}{2\sigma^{2}}\right)$$ (1)

Where is used to adjust smoothness and is set as the distance with the *k*-th neighbor (like K-Nearest Neighbor) in the distance matrix *d*_*ij*_. *k* was set as the default value (default 15) in *scanpy* package. ***A***_*n*×*n*_ was used in the message passing process of GAE.

### 2.2 Graph autoencoder

Topological information between cells can be obtained by GAE [23]. In a classical GAE, the encoder is composed of the GNN layer to produce node embeddings, and the decoder is trained to reconstruct the adjacency matrix by producing a soft reconstruction matrix. In this work, Graph convolutional network (GCN) [25] was used for the unit of encoder, the update of cell embedding ***H***^*l*^ in the *l*^*th*^ layer follows:

$$H^{l}=\tanh\left(\tilde{A}H^{l-1}W_{l-1}\right)$$ (2)

Where $\tilde{A}={\hat{D}}^{-\frac{1}{2}}A{\hat{D}}^{-\frac{1}{2}}$ is the symmetrically normalized adjacency matrix and $\hat{D}$ is the degree matrix of ***A***. Tanh is a hyperbolic tangent activation function $\left(\frac{e^{x}-e^{-x}}{e^{x}+e^{-x}}\right)$. ***W***_*l*-1_ is the learnable parameter of each encoder layer.

A dot product reconstruction (3) was used to constitute the decoder. The reconstructed adjacency matrix $\bar{A}$ is obtained from the outputs of encoder ***H***_***l***_. Finally, a mean square error (MSE) between reconstruction graph and original graph was designed for loss function (4).

$$\bar{A}=Sigmoid\left(H_{l}\cdot H_{l}^{T}\right)$$ (3)

$$L_{graph}=MSE(\bar{A},A)=∥\bar{A}-A{∥}^{2}$$ (4)

Where $moid(x)=\frac{1}{1+e^{-x}}$.

### 2.3 Denoising autoencoder

ZINB or NB distribution is one of the approximate distributions of scRNA-seq data [16]. ZINB-based autoencoder has been applied to extract denoising embedding and overcome the discrete property and over-dispersion of scRNA-seq dataset:

$$P_{NB}\left(R_{n\times m}^{\prime }|,\right)=\frac{\Gamma \left(R_{n\times m}^{\prime }+\theta \right)}{\Gamma \left(R_{n\times m}^{\prime }+1\right)\Gamma (\theta )}\times {\left(\frac{\theta }{\theta +\mu }\right)}^{\theta }\times {\left(\frac{\mu }{\theta +\mu }\right)}^{X_{ij}}$$ (5)

$$P_{ZINB}\left(R_{n\times m}^{\prime }|\pi ,\mu ,\theta \right)=\pi \delta_{0}\left(R_{n\times m}^{\prime }\right)+(1-\pi )\times P_{NB}\left(R_{n\times m}^{\prime }|\mu ,\theta \right)$$ (6)

Where ${R^{\prime }}_{n\times m}$ is defined in the process of data preprocessing to represent the raw counts corresponding to the input matrix. Mean (***μ***), dispersion (***θ***) is the parameter of negative binomial distribution and is the probability that a zero in the raw count is a dropout event.

The encoder accepts ***X***_*z-score*_ as input and its output layer produces reduced-dimension latent embedding ***Z***. The decoder subnetwork, on the contrary, takes ***Z*** as input and produces reconstructions of the original input ***X***_*z-score*_. The encoder and decoder are both Multilayer Perceptron (MLP)-based neural networks. Then, three different fully connected layers are used respectively for fitting mean (***μ***), dispersion (***θ***) and the dropout probability (***π***):

$$\begin{array}{l} Z=W_{encoder}\left(X_{z}{}_{-score}+e\right) \end{array}$$ (7)

$$D=W_{\text{decoder}}(Z)$$ (8)

$$\pi =Sigmoid\left(DW_{\pi }\right)$$ (9)

$$\mu =Diag\left[exp\left(DW_{\mu }\right)\right]$$ (10)

$$\theta =exp\left(DW_{\theta }\right)$$ (11)

Where *e* represents the random Gaussian noise (0 mean and 0.01 variance). The three different fully connected layers are nonlinear activated by diverse ways. The dropout probability (***π***) should be in a reasonable scale (0–1), so a sigmoid is used for standardization. The mean (***μ***) and dispersion (***θ***) are non-negative values, hence exponential function is chosen as activation function. In order to prevent overfitting, the mean (***μ***) is normalized to the same extent with the raw count size for each cell (i.e., *Diag* [] operation). All the ***W*** represent the learnable parameters. Finally, the loss function of DAE is the negative log of the ZINB likelihood:

$$L_{zinb\;}(\pi ,\mu ,\theta |X)=log\left(P_{ZINB}\left(R_{n\times m}^{\prime }|\pi ,\mu ,\theta \right)\right)$$ (12)

### 2.4 Information fusion block

Attention mechanism has shown its effectiveness in natural language processing and computer version [26, 27]. In the attention layer, a global attention score will be calculated to guide the feature extraction based on correlation among samples. Therefore, we construct an information fusion block to merge the denoising embedding and topological embedding through the multi-head attention mechanism:

$$Q_{M}^{l}=W_{M}^{q}H^{l},K_{M}^{l}=W_{M}^{K}E^{l},V_{M}^{l}=W_{M}^{V}E^{l}$$ (13)

$$\text{Attention}\;\text{score}:\;a_{M}^{l}=softmax\left(Q_{M}^{l}\cdot K_{M}^{l}\right)$$ (14)

$$\text{Output}:R^{l}=W\cdot Concat\left(a_{1}^{l}\cdot V_{1}^{l},a_{2}^{l}\cdot V_{2}^{l},\ldots ,a_{M}^{l}\cdot V_{M}^{l}\right)+E^{l}$$ (15)

Where the query $Q_{M}^{l}$ is calculated by the topological embedding ***H***^*l*^ from GAE, and the key $K_{M}^{l}$, value $V_{M}^{l}$ are calculated by the denoising embedding ***E***^*l*^ from DAE. The attention score is calculated by the dot-product attention mechanism. *M* represents the number of heads. All the ***W*** and ***W***_***M***_ represent the learnable parameters. In AttentionAE-sc, two information fusion blocks are adopted so two outputs ***R***^*l*^ are calculated successively by the *l*^*th*^ embedding of GAE and DAE. The output ***R***^1^ is used as the input of next encoder layer (fully connected layers followed by the ReLU activation function), and the output ***R***^2^ is used as the feature representation (embedding ***Z***) of clustering stage.

### 2.5 Deep embedding clustering

In the clustering stage, DEC [17] is applied to carried out a self-optimizing soft clustering. The soft clustering distribution Q is defined as the probability of cell belonging to each cluster center measured by student’s t distribution:

$$q_{ij}=\frac{{\left(1+||z_{i}-\kappa_{j}|{|}^{2}\right)}^{-1}}{\sum_{k}{\left(1+||z_{i}-\kappa_{k}|{|}^{2}\right)}^{-1}}$$ (16)

Where *k*_*j*_ (j = 1, 2, …, k) is the cluster centers, obtained by Leiden algorithm [15] with pre-trained embedding ***Z*** as input. The optimized high-confidence distribution P is constructed as following:

$$p_{ij}=\frac{q_{ij}^{2}/\sum_{i=1}^{n}q_{ij}}{\sum_{c=1}^{k}(q_{ic}^{2}/\sum_{i=1}^{n}q_{ic})}$$ (17)

The optimization process of clustering is defined as the KL divergence between the soft clustering distribution Q and the target distribution P:

$$L_{kl-loss}=KL(P∥Q)=\sum_{i}\sum_{j}p_{ij}\;log\;\frac{p_{ij}}{q_{ij}}$$ (18)

In summary, the total loss function is:

$$train:\;L_{train}=L_{zinb}+r_{1}L_{graph}$$ (19)

$$finetuning:\;L_{fit}=L_{kl-loss}+r_{2}L_{train}$$ (20)

Where *r*_1_ and *r*_2_ are the factors to balance multi-object optimization (default 0.1). The clustering process is only carried out in the fine-tuning stage. AttentionAE-sc was implemented in Python 3.8. Dimensions of all hidden layers were set as 256, 64, 16, 64, 256. Two multi-head attention blocks were used, and the number of heads was 8. In order to reduce the time cost of model training, a dimension reduction linear layer was shared by DAE and GAE. In the process of model training and fine-tuning, Adam was used as the optimizer. The learning rate was 0.001 and a gradient clipping with L2 norm maximum of 3 was adopted. In the fine-tuning stage, training will be stopped prematurely if the number of altered labels is fewer than 1/1000.

## 3. Results

### 3.1 Evaluation metric and baseline methods

In this section, we tested AttentionAE-sc on 16 scRNA-seq datasets [28, 29, 30, 31, 32, 33] from different organs or platforms (containing 870–9552 individual cells or 4–11 cell groups). Detailed information was presented in Table 1. We used the labels provided by these datasets as the ground truth and adjusted Rand index (ARI) [34] and normalized mutual information (NMI) [35] were used as metrics for evaluation of model performance. The similarity between the predicted labels and the ground truth was compared from different points of view. The higher the value is, the more accuracy the clustering results are. When multiple methods employ the same clustering strategy, such as community detection, their performance in clustering distribution can be evaluated by calculating the internal metrics like the silhouette score [36] and Davies-Bouldin score [37]. For internal metric, the better the clustering performance is, the greater the distance between predicted clusters and the closer the distance within each cluster; this results in a larger silhouette score and a smaller Davies-Bouldin score.

**Table 1:** General information of 16 scRNA-seq datasets used for methods evaluation.

Index	Dataset Name	Number of Cell	Cell groups	Platform
1	Muraro	2122	9	CEL-seq2
2	Quake 10x Bladder	2500	4	10 X Genomics
3	Quake 10x Limb Muscle	3909	6	10 X Genomics
4	Quake 10x Spleen	9552	5	10 X Genomics
5	Quake Smart-seq2 Diaphragm	870	5	Smart-seq2
6	Quake Smart-seq2 Limb Muscle	1090	6	Smart-seq2
7	Quake Smart-seq2 Lung	1676	11	Smart-seq2
8	Quake Smart-seq2 Trachea	1350	4	Smart-seq2
9	Romanov	2881	7	Illumina HiSeq
10	Baron Pancreas 1	1937	14	inDrop
11	Baron Pancreas 2	1724	14	inDrop
12	Baron Pancreas 3	3605	14	inDrop
13	Baron Pancreas 4	1303	14	inDrop
14	Baron Pancreas mouse	1886	13	inDrop
15	Chung	515	5	inDrop
16	Klein	2717	5	Illumina HiSeq

We selected several state-of-art scRNA-seq clustering methods as baseline methods, including DESC [18], Leiden (scanpy) [4, 15], SC3 [13], scGNN [10], scGAC [11], graph-sc [21], scDeepCluster [9], scvi-tools [20], and SCEA [24]. Among these methods, scGAC, scDeepCluster and SCEA need to specify the number of clusters when using K-means algorithm to initialize the cluster centers. Both K-means and Leiden are supported by graph-sc, but we adopted the results obtained by Leiden algorithm considering that methods without setting the number of cell groups were more suitable for unsupervised scRNA-seq clustering analysis. In the case of SC3, we employed the Tracy-Widom theory on random matrices to estimate the optimal number of clusters before executing all steps of SC3 analysis. See S1 Table for additional details of baseline methods.

### 3.2 AttentionAE-sc achieved excellent clustering results

**Fig 2:**
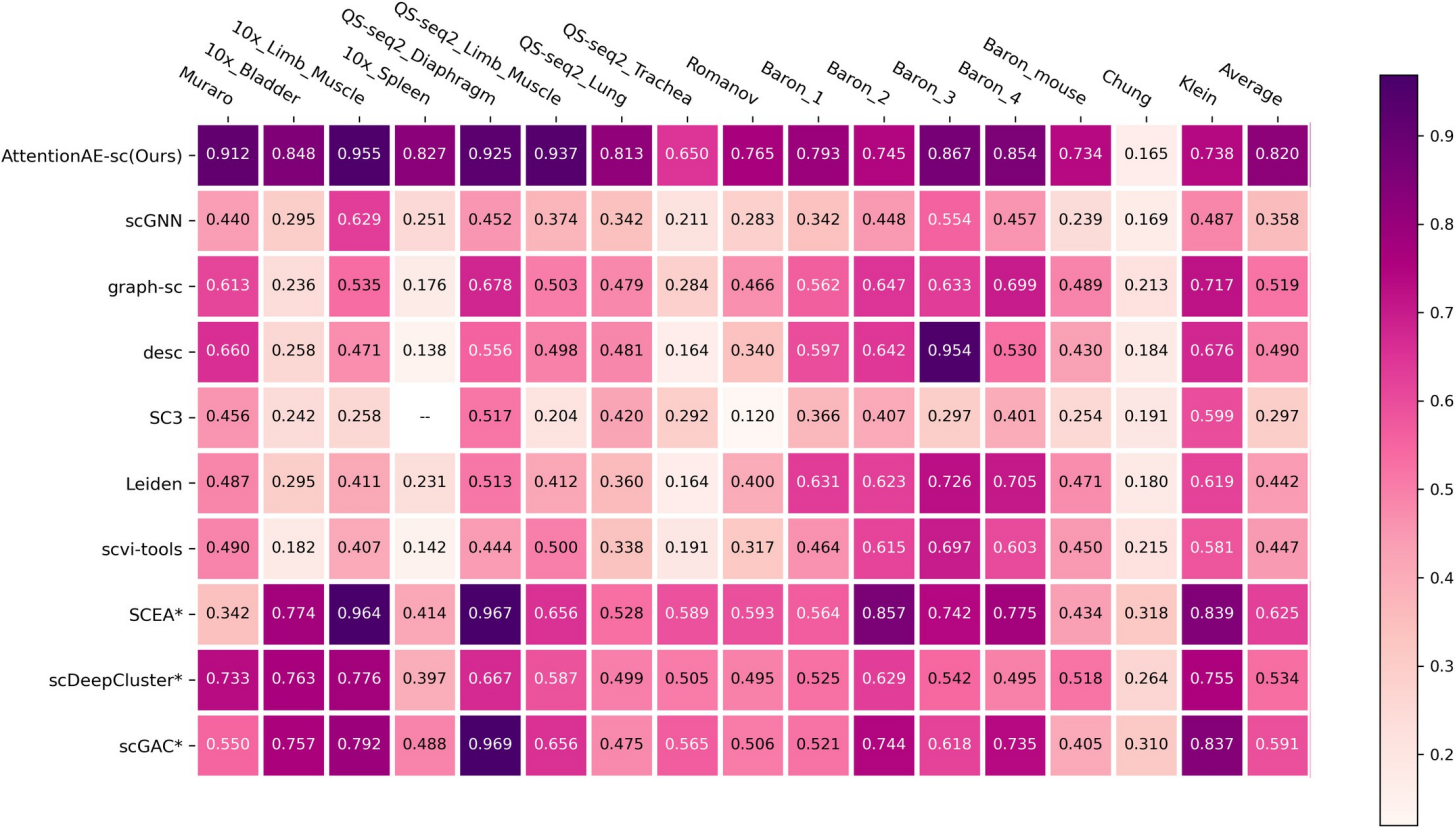
ARI score of AttentionAE-sc (our methods) and all baseline methods in 16 scRNA-seq datasets. Arithmetic mean was taken as results of each dataset after running each method five times under different random seeds. Methods that need to specify the number of clusters were marked with an asterisk (*).

The experimental results were shown in Figs 2 and S2. On 12 real scRNA-seq datasets, the performance of AttentionAE-sc was at the forefront. On other real scRNA-seq datasets, the performance of AttentionAE-sc came out in front and completely better than the community detection-based methods (scGNN, graph-sc, DESC, Leiden, scvi-tools). Like these methods, AttentionAE-sc adopted the same strategy to avoid manually setting the number of predicted clusters and achieved excellent results, about 60%-90% higher ARI score and 15%-36% higher NMI score.

An intriguing observation reveals that the methods relying on a known cell group number yielded better external metric scores when there are fewer actual cell groups, particularly in datasets with fewer than 6 cell groups. In contrast, other methods that tend to partition the datasets into more predicted cell groups. Nonetheless, the overall performance of AttentionAE-sc remained superior to that of the K-means-based method. Among these K-means-based methods, SCEA exhibited better performance. In comparison to scGAC, which also utilized a GAE and a self-optimizing DEC, its performance was less satisfactory across most datasets. To scDeepCluster, a classical ZINB-based model, AttentionAE-sc obtained better results because of leveraging the interaction among cells during the process of learning cell representations. By effectively combining the fundamental frameworks of these two approaches, AttentionAE-sc aquired outstanding results. However, SC3 did not perform as well as deep learning-based methods, often overestimating the cluster count when using random matrices and producing null values with larger datasets (10x spleen). In general, AttentionAE-sc achieved a competitive performance without specifying the number of clusters.

**Fig 3:**
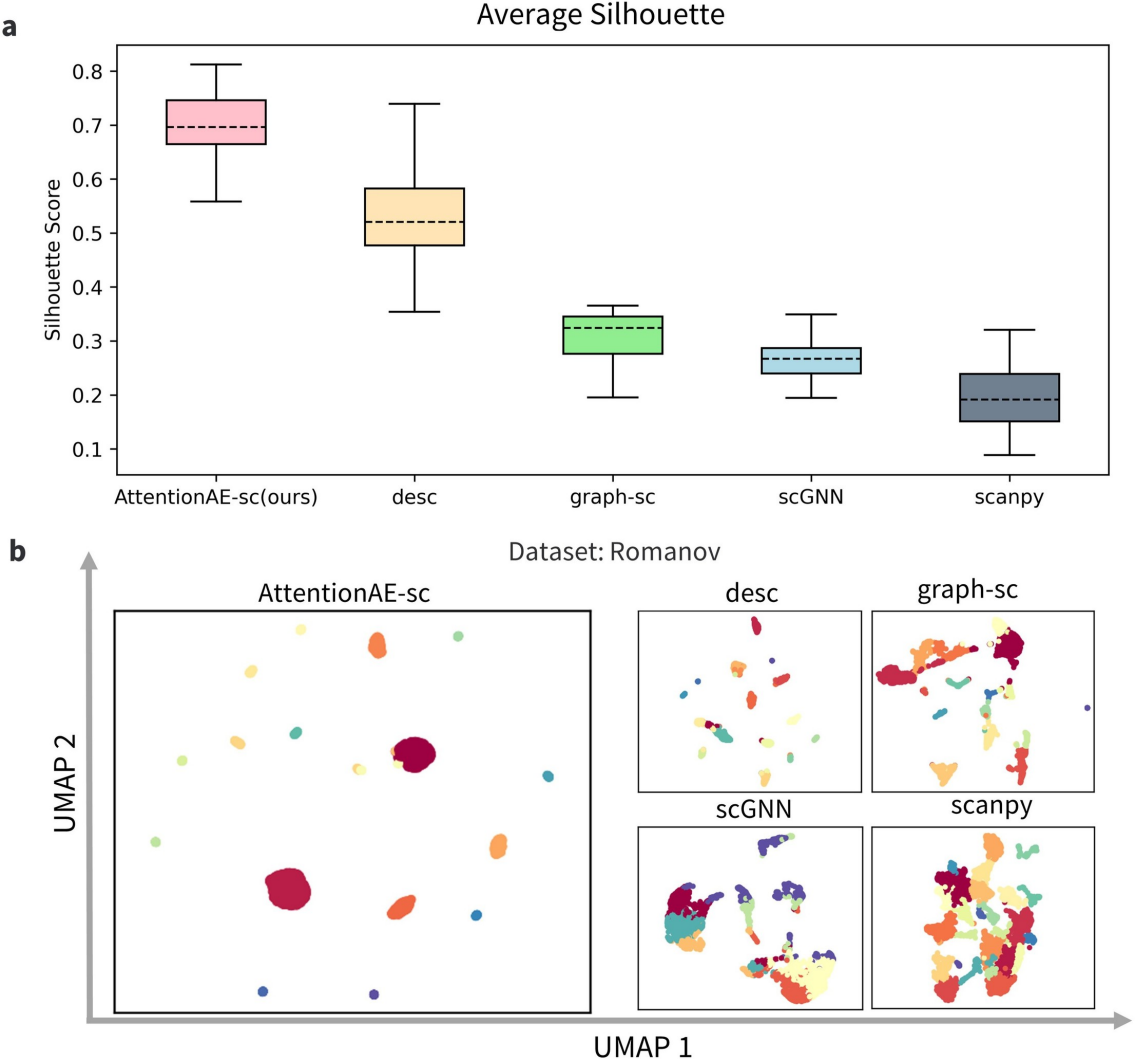
Evaluations of cell embedding. **a.** Clustering performance of AttentionAE-sc and four methods based on community detection algorithm using Silhouette scores as metrics. Each box contains the results of 16 datasets (run 5 times by different random seeds). In the box diagram, the dotted line represents the median score, and the upper or lower solid lines represent the maximum or minimum score. **b.** Comparison of UMAP visualization from different methods on the dataset Romanov. AttentionAE-sc learned more clustering-friendly embedding for single-cell clustering.

To analyze the quality of cell representation for clustering, we visualized the latent embeddings of AttentionAE-sc and used the internal metrics (S2B and S2C Fig) as the measurement of the clustering performance. Compared with other 4 state-of-art community detection-based methods (Fig 3A), AttentionAE-sc achieved the best performance. In the baseline methods, graph-sc and scGNN, like AttentionAE-sc, also employed GAE for feature extraction, but they acquired a suboptimal performance due to splitting representation learning and clustering into separate tasks. By contrast, DESC and AttentionAE-sc obtained the higher silhouette score because the soft clustering strategies were adopted to simultaneously optimize feature extraction and clustering allocation, but AttentionAE-sc further considered the similarity among cells and directly applied topological embedding to construct the cell representation through a fusion feature attention layer. In addition, the Leiden algorithm (based on scanpy) obtained a lower performance with raw gene expression matrices as input. We can intuitively perceive the advantages of embedding learned by AttentionAE-sc through UMAP visualization (Fig 3B). AttentionAE-sc obtained the larger distinction between each group and the degree of internal tightness. S3 Fig showed the UMAP visualization of other scRNA-seq datasets, which was more intuitive to reflect the clustering and representation performance of those 5 methods including AttentionAE-sc.

### 3.3 AttentionAE-sc constructed better relationships among cells

**Fig 4:**
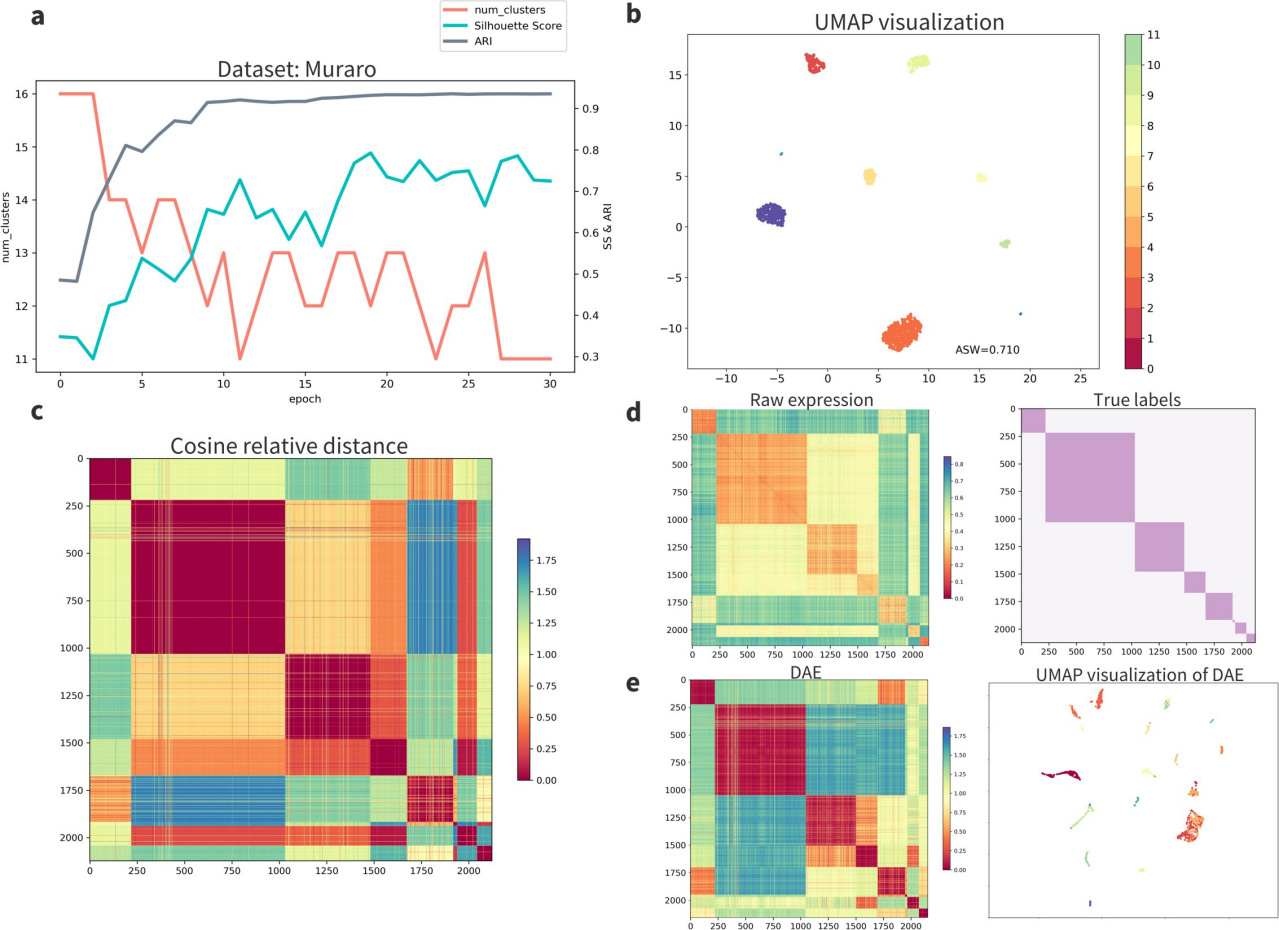
AttentionAE-sc construction better relationships among cells (Muraro). **a.** The trend of the number of cell groups predicted and ARI scores changing with training epochs during the clustering stage. The better clustering performance was obtained, when the cluster centers were adaptively chosen with each iteration and redundant cluster centers were discarded. Other datasets were shown in S4 Fig. **b.** The visualization of cell embeddings. **c.** the heatmap of relative cosine distance between cells calculated by the embeddings of multi-head attention layer. **d.** the heatmap of relative cosine distance between cells calculated from the input expression matrix. On the right subfigure, we sorted the dataset follow per cell groups to get an intuitive visualization of among cells distance (c, d, e). **e.** the heatmap of relative cosine distance between cells calculated by ordinary denoising autoencoder (DAE) and the corresponding visualization.

AttentionAE-sc constructed relationships among cells through the attention-based information fusion blocks. In each iteration of the clustering stage, cells can receive the information from other cells according to the attention mechanism, so that the cluster centers were adaptively chosen and redundant cluster centers were discarded (Figs 4A and S4), since similar cells were closer in the hidden space and were easier to be assigned to the same cluster. For verification, we took the outputs of information fusion blocks as dimension reduction features to calculate the relative distance between cells and estimated the influence of information fusion block based on the assumption that the output of attention layer can retain the relationships among cells extracted by AttentionAE-sc. Comparing the relative distance visualization of AttentionAE-sc (Fig 4C) with raw expression matrix (Fig 4D) and features extracted without information fusion block (Fig 4E), it’s clear to observe that the cells distribution in the same groups were closer and were more distinctive between the different groups based on the embeddings learned by AttentionAE-sc.

To gain further insights into the interpretability of the attention blocks, we visualized the weight of attention neural network directly, which represent the attention scores between each pair of cells (S5 Fig). It becomes evident that similar global attention information was consistently transmitted among cells of the same cell types. Consequently, this reaffirms the consistent results observed in Fig 4C. From another perspective, each attention head seemed to focus on learning the attention scores of specific subsets of cells, indicating that multi-head attention mechanism can effectively capture the distinctions between cell groups at the transcriptome scale. In summary, AttentionAE-sc learned a more clustering-friendly cell representation and deserved a great clustering result with the iterative clustering process.

### 3.4 AttentionAE-sc exhibited great stability and robustness

We tested the robustness and stability of AttentionAE-sc by input manual dropout and down-sampling. For the dropout experiment, 10% - 50% gene expression values from the real scRNA-seq datasets were randomly manipulated as zero values and the impaired gene expression matrix were adopted to compare the clustering performance of AttentionAE-sc with DAE. For the down-sampling experiment, 20% - 80% cells from the real scRNA-seq datasets were sampled in a random and stratified way to construct the clustering model, that was used to decide the cell subpopulations for whole datasets. As shown in S6 Fig, AttentionAE-sc was more capable of handling impaired datasets than the ordinary DAE-based methods (S6A Fig). In addition, selecting a part of sampled cells at random achieved comparable performance to that of using the entire datasets (S6B Fig).

### 3.5 Model ablation experiment

**Fig 5:**
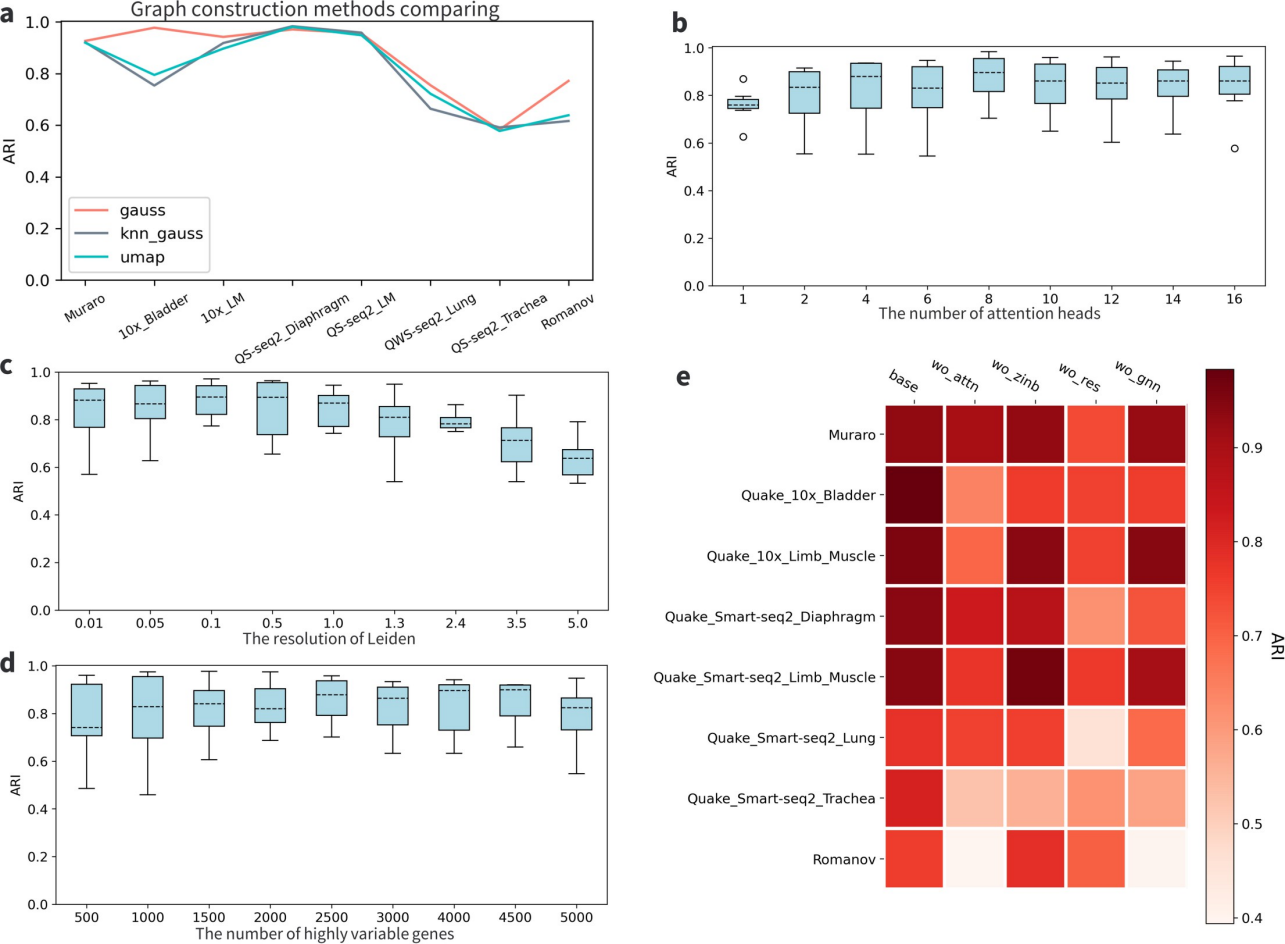
Hyperparameters search and the ablation experiment. **a.** Comparison of different methods for cell connectivity calculations. When compared to the UMAP method, the ’gauss’ method demonstrated superior performance. **b.** Comparison of different settings for the number of attention heads. **c.** Comparison of different settings for the resolution parameter in the Leiden algorithm. **d.** Comparison of different settings for the number of highly variable genes. **e.** Model ablation experiment of AttentionAE-sc in 8 scRNA-seq datasets. Four conditions were tested, including the absence of the information fusion block (wo attn), the absence of the ZINB loss function (wo zinb), the absence of residual connections (wo res), and the absence of GAE (wo gnn).

We conducted a hyperparameter search to establish a baseline model prior to conducting the ablation experiment. To construct the cell-cell graph, cell connectivity was calculated using a Gaussian kernel following the diffusion maps [38] with adaptive width [39]. The computational process was implemented using the scanpy package [4]. While we explored other methods integrated into the package, such as the UMAP [40], it was consistently evident that the ‘gauss’ method outperformed others (Fig 5A). Other hyperparameters that significantly impact the performance of AttentionAE-sc include the number of attention heads in the information fusion blocks, the resolution parameters in the Leiden algorithm, and the number of highly variable genes (Figs 5B–5D and S7). The optimal choice for the number of attention heads is 8. Having too few attention heads results in lead inadequate learning of cell interactions, whereas an excessive number leads to redundancy in model parameters. The resolution of Leiden primarily governs the number of clusters generated by the algorithm. Smaller resolution values yield a greater number of distinct cell types. Our results indicate that optimal performance falls within the range of 0.1 to 1. Therefore, we selected a resolution of 1 for our experiments. Highly variable genes, characterized by their significant variance within the datasets, are typically considered candidate genes for differential expression analysis. Consequently, before conducting single-cell data clustering, it is advisable to preselect a subset of highly variable genes as features. Our findings suggest that utilizing 2,500 highly variable genes yields the best results, as too few or redundant features can negatively impact the clustering outcomes.

To determine the importance of each component in AttentionAE-sc, we compared the performance of AttentionAE-sc without attention-based information fusion block (wo attn), without ZINB loss function (wo zinb), without residual connection (wo res) and without GNN (wo gnn). As shown in Figs 5E and S8, the addition of information fusion block can significantly improve the clustering performance. However, when the attention layers were substituted with a multi-layer perceptron, performance deteriorated. Besides, it’s worth noted that AttentionAE-sc can achieve comparable results with ZINB-based DAE in several datasets when a L2 norm-based DAE (wo zinb) was used, so AttentionAE-sc can also perform well without using the approximation assumption of ZINB distribution. Nevertheless, the hypothesis of the ZINB distribution can enable the model to adapt to a broader range of datasets and enhance its generalization capability. Furthermore, constructing residual connections in front of the attention layer typically expedites model convergence and mitigates the risk of overfitting. Poor model performance was observed when denoising embedding wasn’t connected to the representation of cell (wo res). Finally, we conducted a test without the use of GNN, where the keys and values of the attention layer were derived from the embedding obtained from the DAE. The results demonstrated that the absence of GAE-based learning of cellular interactions leaded to a decline in model performance. As a result, we made the inference that excellent performance of AttentionAE-sc was attributed to the information fusion block which can learn the relation among cells iteratively to obtain a clustering-friendly representation.

### 3.6 Experiment in BRCA dataset

**Fig 6:**
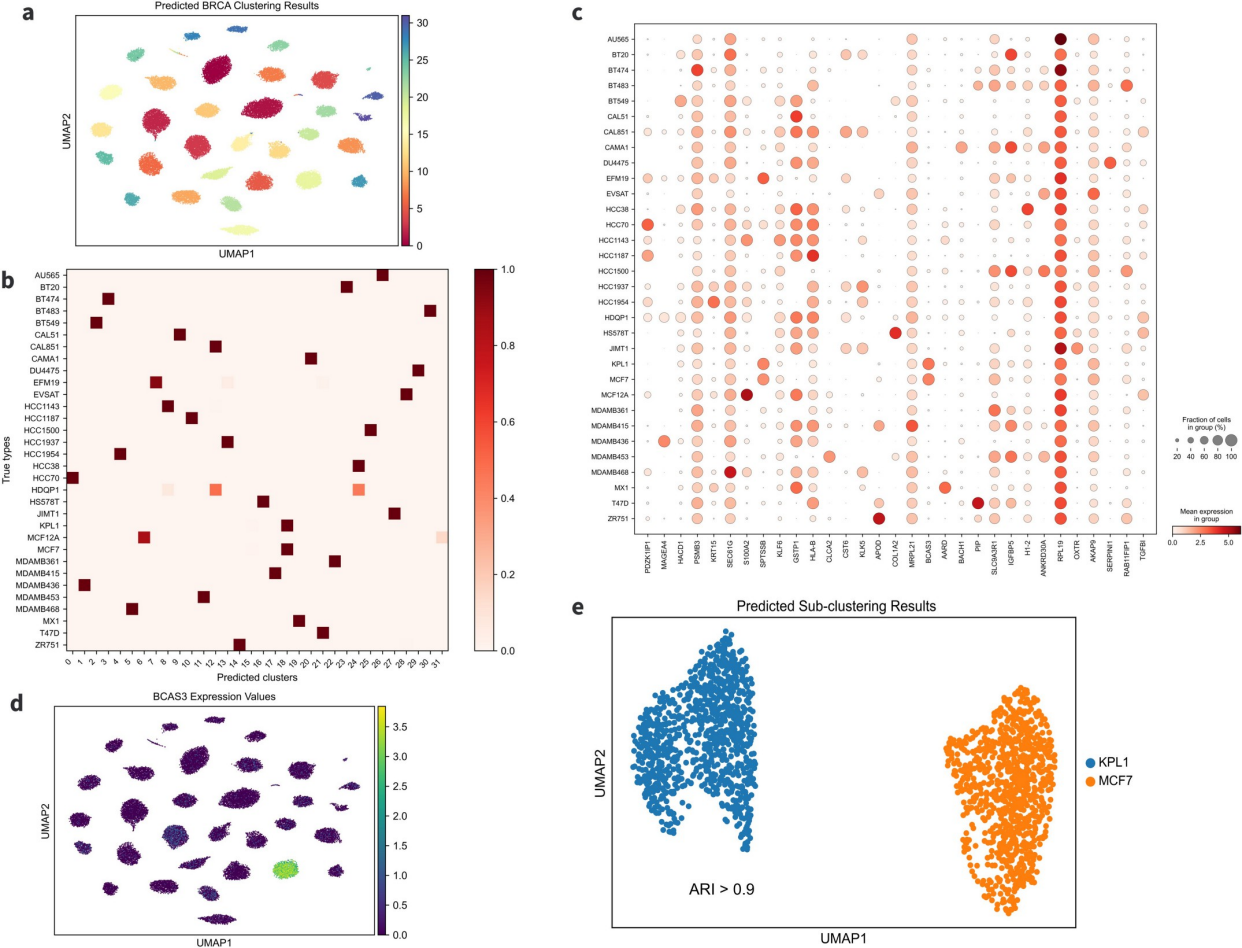
Experiments in BRCA dataset. **a.** UMAP visualization of the clustering results on the BRCA datasets. **b.** Comparison of cell distribution between the predicted clusters and the ground truth cell lines. In the heat map, each row represents a class of cells from the same cell line, and each column represents a class of cells from the same predicted cluster. There was generally a one-to-one correspondence between cell lines and predicted clusters for ideal clustering results. **c.** Expression dot plot of identified DEGs in different cell lines. Based on the predicted cell labels, DEGs of all clusters were calculated by the Wilcoxon test (cluster 15 only contains 30 cells, so it was excluded). Top 1 DEGs of predicted clusters were selected as the potential marker genes. **d.** The pot plot was drawn to visualize the expression in each cell line and the most of cell lines can be distinguished obtained DEGs. *BCAS3* was specifically expressed in cluster 18, i.e. MCF7 and KPL1. **e.** The UMAP visualization of the sub-clustering results on the cluster 18 by AttentionAE-sc, which was consist of these two cell lines.

To further evaluate the performance of AttentionAE-sc, we conducted an analysis on a breast cancer (BRCA) single-cell atlas [41]. This dataset consists of 35,276 single cells derived from 32 cell lines. The results of our analysis were presented in Figs 6 and S9. After receiving the predicted cluster labels and the cell representation according to AttentionAE-sc, we further obtained two-dimensional visual results based on UMAP (Fig 6A). The predicted clusters closely match the ground truth (Fig 6B) and the vast majority of cells from the same cell line were assigned to the same class. Additionally, through the differential expression analysis between the predicted clusters, we identified a set of differentially expressed genes (DEGs) that can serve as marker genes. These marker genes effectively distinguish cells to the corresponding cell line types (Fig 6C).

However, some extra clustering assignment results have emerged. For instance, cells from different cell lines, KPL1 and MCF7, have been predicted to the same groups (cluster 18). One of the identified DEG, *BCAS3*, has been found to be a biomarker for BRCA [42, 43]. Interestingly, *BCAS3* exhibits similar specific expression in both of these two cell lines (Fig 6D). In addition, it has been observed in several studies that KPL1 is a sub-clonal cell line derived from MCF7 [44, 45]. Subsequently, we proceeded to perform sub-clustering within cluster 18, and observed a striking separation of cells from these two cell lines (Fig 6E). To investigate the variation in AttentionAE-sc’ s ability to distinguish cells from different scale datasets, we analyzed the differences of input features (S9A Fig). Indeed, a major difference in the clustering stage and sub-clustering stage can be attributed to the selection of genes, which are influenced by the proportion of cells of interest in the dataset. When the same feature screening method was used, we may obtain different initial input features in different stages, leading to varying results. We argue that the limited number of cells from the cell line HDQP1 in the BRCA dataset is the reason for their scattered prediction in the clustering process. Additionally, the only non-cancer cell line MCF12A was divided into two groups (cluster 6 and cluster 31). One possibility is that they are different cell subtypes. Therefore, we further analyzed the expression heterogeneity between these two groups. Based on the Wilconxon test, we calculated the DEGs for each group (p-value <0.05, listed in S2 Table) and conducted KEGG enrichment analysis (S9B Fig) [46]. The preliminary findings indicate that there is a significant difference in the cell states between the two subtypes. In cluster 31, pathways associated with inflammation and apoptosis are up-regulated, while pathways related to cell proliferation are down-regulated. In conclusion, AttentionAE-sc proves to be effective in distinguishing cells within multi-scale datasets and provides valuable insights into the heterogeneity among different cell subtypes.

## 4. Discussion

In this work, we propose AttentionAE-sc, a novel deep learning-based scRNA-seq clustering method, relying on an attention-based information fusion block, which combines the denoising autoencoder with the graph autoencoder to learn a clustering-friendly cell representation. It can not only deal with sparsity and dropout events of scRNA-seq datasets by ZINB-based autoencoder, but also directly considers the relationship among cells to guide the dimension reduction and get a clustering-friendly representation. Therefore, AttentionAE-sc can easily extract denoising features from the sparse scRNA-seq data, and the ideal clustering partition is obtained without specifying the number of groups.

AttentionAE-sc acquired an excellent performance on 16 scRNA-seq datasets, with an average of 60% ARI score and 18% NMI score higher than 9 baseline methods. Compared with these state-of-art methods, AttentionAE-sc achieved an exceptional performance, including a high internal metric silhouette score that reflects the quality of cell representations. Furthermore, we explored the relationship between the outstanding clustering results and the clustering-friendly cell representation obtained during the optimizing clustering stage. We also analyzed the stability and robustness of AttentionAE-sc and examined the contribution of each module component. Moreover, experiments conducted on BRCA datasets demonstrated the excellent performance of AttentionAE-sc in clustering analysis and its capability to unveil the biological significance within scRNA-seq datasets.

However, AttentionAE-sc as well as some other methods [10, 11] aims to construct a more elaborate model by taking cell interactions into account but is at the cost of computation time (S3 Table). In addition, in each iteration of the optimized clustering process, AttentionAE-sc takes all samples as input which limits its usage in a more large-scale dataset. Moreover, existing annotated single-cell datasets [47, 48] possess the potential to construct pre-training models and reduce the training difficulties but weren’t fully utilized. Concurrently, the incorporation of prior knowledge, which aids in establishing cellular relationships [49], holds promise as valuable information for constructing deep learning models [50]. In the future, it’s still a challenge to propose a canonical single-cell clustering pipeline. We will explore more efficient clustering strategies which can make full use of increasing annotated or unannotated single-cell datasets.

## Supporting information

S1 Fig The architecture of attention-based fusion block. Different embeddings from DAE and GAE are combined by a multi-head attention layer and residual connection is adopted to promote the training efficiency of DAE module.
(TIF)

S2 Fig The clustering performance of AttentionAE-sc and baseline methods in 16 scRNA-seq datasets. **a**. NMI scores of AttentionAE-sc and 9 baseline methods. **b**. Silhouette scores of 6 community detection-based methods (including AttentionAE-sc). **c**. Davies-Bouldin score of 6 community detection-based methods (including AttentionAE-sc). Arithmetic mean is taken as results of each dataset after running each method five times under different random seeds. Methods that need to specify the number of clusters are marked with an asterisk (*).
(TIF)

S3 Fig Comparison of UMAP visualizations from AttentionAE-sc and 4 community detection-based state-of-art methods on the 16 scRNA-seq datasets. The 5 subfigures in each row are the cell projections obtained from the 2-dimentional UMAP features, which are calculated from the cell representation obtained by the different methods. Each dataset consists of 2 rows of subfigures that adopt the ground truth or predicted labels by different method respectively (different color clusters in the subfigure). In summary, AttentionAE-sc obtained the larger distinction between each group and the degree of internal tightness and the cell annotation is closer to the ground truth.
(TIF)

S4 Fig The variation trend of the number of cell types predicted and the result scores in the clustering stage of 15 scRNA-seq datasets (Muraro was shown in Fig 4A). (TIF)

S5 Fig The visualization of the weight of attention blocks. Each head contains a *n* × *n* matrix (*n* is the number of cells), which represents the attention scores between each pair of cells. (TIF)

S6 Fig Model stability and robustness test in scRNA-seq datasets. **a.** The ARI score of clustering results with random manual dropout rate of gene expression values set as 10%, 20%, 30% and 50% in the scRNA-seq datasets. All experiments run 5 times with different random seeds. Base (the pink box) is the performance of AttentionAE-sc without manual dropout. Compared AttentionAE-sc (drop 10 to drop 50, the blue box) with ordinary DAE (DAE 10 to DAE 50, the grey box), AttentionAE-sc is less influenced. **b.** The ARI score of clustering results with random and stratified input down-sampling rate of cells set as 20%, 40%, 60% and 80% respectively in the scRNA-seq datasets. All experiments run 5 times with different random seeds. Base (the pink box) is the performance of AttentionAE-sc with the whole scRNA-seq datasets as input. The average score is displayed below the x label on each box.
(TIF)

S7 Fig Hyperparameter search experiment. Three hyperparameters significantly influence the clustering performance of AttentionAE-sc: the number of attention heads in the information fusion blocks (**a**), the resolution parameters in the Leiden algorithm (**b**), and the number of highly variable genes (**c**).
(TIF)

S8 Fig Model ablation experiment of AttentionAE-sc in 8 scRNA-seq datasets. NMI, silhouette score (ASW) and Davies-Bouldin score (DB) are used as metrics. Four conditions were tested, including the absence of the information fusion block (wo attn), the absence of the ZINB loss function (wo zinb), the absence of residual connections (wo res), and the absence of GAE (wo gnn).
(TIF)

S9 Fig Visualization of the experiments in the BRCA dataset. **a.** Venn diagram comparing the difference of highly variable genes after the preprocessing process between original clustering stage and sub-clustering stage. b. The results of KEGG enrichment analysis in the cluster 6 and cluster 31 based on the acquired DEGs in the S2 Table. In the histogram, the dotted line represents a threshold that the corrected p-value is 0.05 and the higher scores indicates significant enrichment.
(TIF)

S10 Fig Identification of Rare Cell Types. To assess the capacity for recognizing rare cell types, we opted to visualize cell types with a limited number of cells in the datasets. The results demonstrate that AttentionAE-sc possesses the capability to predict clusters with a small number of cells and distinguish rare cell types.
(TIF)

S1 Table General information of 9 state-of-art baseline methods. Overview contains code source and operational environment for experiment in the real scRNA-seq datasets.
(DOCX)

S2 Table Differentially expressed genes between cluster 6 and cluster 31 in the breast cancer single-cell atlas experiment. For each clustering, the threshold for considering the presence of differential expression is 0.05 (P-values in Wilcoxon test).
(DOCX)

S3 Table Elapsed time (sec) in the train process. Compare with another GNN-based methods in the 14 real scRNA-seq datasets. Nvidia RTX 3070Ti (8 GB RAM) was used in the gpu test.
(DOCX)

## References

[1] Shapiro E., et al. Single-cell sequencing-based technologies will revolutionize whole-organism science. Nat. Rev. Genet. 2013, 14, 618–630. doi: 10.1038/nrg3542 23897237

[2] Kolodziejczyk A.A., et al. The technology and biology of single-cell RNA sequencing. Mol. Cell 2015, 58, 610–620. doi: 10.1016/j.molcel.2015.04.005 26000846

[3] Satija R., et al. Spatial reconstruction of single-cell gene expression data. Nat. Biotechnol. 2015, 33, 495–502. doi: 10.1038/nbt.3192 25867923 PMC4430369

[4] Wolf F.A., et al. SCANPY: large-scale single-cell gene expression data analysis. Genome Biol. 2018, 19, 15. doi: 10.1186/s13059-017-1382-0 29409532 PMC5802054

[5] Lahnemann D., et al. Eleven grand challenges in single-cell data science. Genome Biol. 2020, 21, 31. doi: 10.1186/s13059-020-1926-6 32033589 PMC7007675

[6] Svensson V., et al. Power analysis of single-cell RNA-sequencing experiments. Nat. Methods 2017, 14, 381–387. doi: 10.1038/nmeth.4220 28263961 PMC5376499

[7] Kiselev V.Y., et al. Challenges in unsupervised clustering of single-cell RNA-seq data. Nat. Rev. Genet. 2019, 20, 273–282. doi: 10.1038/s41576-018-0088-9 30617341

[8] Qi R., et al. Clustering and classification methods for single-cell RNA-sequencing data. Brief. Bioinform. 2020, 21, 1196–1208. doi: 10.1093/bib/bbz062 31271412 PMC7444317

[9] Tian T., et al. Clustering single-cell RNA-seq data with a model-based deep learning approach. Nat. Mach. Intell. 2019, 1, 191–198.

[10] Wang J., et al. scGNN is a novel graph neural network framework for single-cell RNA-Seq analyses. Nat. Commun. 2021, 12, 1882. doi: 10.1038/s41467-021-22197-x 33767197 PMC7994447

[11] Cheng Y. and Ma X. scGAC: a graph attentional architecture for clustering single-cell RNA-seq data. Bioinformatics 2022, 38, 2187–2193. doi: 10.1093/bioinformatics/btac099 35176138

[12] Grun D., et al. Single-cell messenger RNA sequencing reveals rare intestinal cell types. Nature 2015, 525, 251–255. doi: 10.1038/nature14966 26287467

[13] Kiselev V.Y., et al. SC3: consensus clustering of single-cell RNA-seq data. Nat. Methods 2017, 14, 483–486. doi: 10.1038/nmeth.4236 28346451 PMC5410170

[14] Levine J.H., et al. Data-Driven Phenotypic Dissection of AML Reveals Progenitor-like Cells that Correlate with Prognosis. Cell 2015, 162, 184–197. doi: 10.1016/j.cell.2015.05.047 26095251 PMC4508757

[15] Traag V.A., et al. From Louvain to Leiden: guaranteeing well-connected communities. Sci. Rep. 2019, 9, 5233. doi: 10.1038/s41598-019-41695-z 30914743 PMC6435756

[16] Eraslan G., et al. Single-cell RNA-seq denoising using a deep count autoencoder. Nat. Commun. 2019, 10, 390. doi: 10.1038/s41467-018-07931-2 30674886 PMC6344535

[17] Xie J., et al. Unsupervised Deep Embedding for Clustering Analysis. arXiv 2016, arXiv:1511.06335. Available from: https://arxiv.org/abs/1511.06335

[18] Li X., et al. Deep learning enables accurate clustering with batch effect removal in single-cell RNA-seq analysis. Nat. Commun. 2020, 11, 2338. doi: 10.1038/s41467-020-15851-3 32393754 PMC7214470

[19] Lopez R., et al. Deep generative modeling for single-cell transcriptomics. Nat. Methods 2018, 15, 1053–1058. doi: 10.1038/s41592-018-0229-2 30504886 PMC6289068

[20] Gayoso A., et al. A Python library for probabilistic analysis of single-cell omics data. Nat. Biotechnol. 2022, 40, 163–166. doi: 10.1038/s41587-021-01206-w 35132262

[21] Ciortan M. and Defrance M. GNN-based embedding for clustering scRNA-seq data. Bioinformatics 2021, 38, 1037–1044. 10.1093/bioinformatics/btab787 34850828

[22] Shao X., et al. scDeepSort: a pre-trained cell-type annotation method for single-cell transcriptomics using deep learning with a weighted graph neural network. Nucleic Acids Res. 2021, 49, e122. doi: 10.1093/nar/gkab775 34500471 PMC8643674

[23] Kipf T.N. and Welling M. Variational Graph Auto-Encoders. arXiv 2016, arXiv:1611.07308. Available from: https://arxiv.org/abs/1611.07308

[24] Abadi S.A.R., et al. An optimized graph-based structure for single-cell RNA-seq cell-type classification based on non-linear dimension reduction. BMC Genomics 2023, 24, 227. doi: 10.1186/s12864-023-09344-y 37127578 PMC10152777

[25] Kipf T.N. and Welling M. Semi-Supervised Classification with Graph Convolutional Networks. arXiv 2017, arXiv:1609.02907. Available from: https://arxiv.org/abs/1609.02907

[26] Vaswani A., et al. Attention Is All You Need. arXiv 2017, arXiv:1706.03762. Available from: https://arxiv.org/abs/1706.03762

[27] Dosovitskiy A., et al. An Image is Worth 16x16 Words: Transformers for Image Recognition at Scale. arXiv 2021, arXiv:2010.11929. Available from: https://arxiv.org/abs/2010.11929

[28] Klein A.M., et al. Droplet barcoding for single-cell transcriptomics applied to embryonic stem cells. Cell 2015, 161, 1187–1201. doi: 10.1016/j.cell.2015.04.044 26000487 PMC4441768

[29] Baron M., et al. A Single-Cell Transcriptomic Map of the Human and Mouse Pancreas Reveals Inter- and Intra-cell Population Structure. Cell Syst. 2016, 3, 346–360 e344. doi: 10.1016/j.cels.2016.08.011 27667365 PMC5228327

[30] Muraro M.J., et al. A Single-Cell Transcriptome Atlas of the Human Pancreas. Cell Syst. 2016, 3, 385–394 e383. doi: 10.1016/j.cels.2016.09.002 27693023 PMC5092539

[31] Romanov R.A., et al. Molecular interrogation of hypothalamic organization reveals distinct dopamine neuronal subtypes. Nat. Neurosci. 2017, 20, 176–188. doi: 10.1038/nn.4462 27991900 PMC7615022

[32] Chung W., et al. Single-cell RNA-seq enables comprehensive tumour and immune cell profiling in primary breast cancer. Nat. Commun. 2017, 8, 15081. doi: 10.1038/ncomms15081 28474673 PMC5424158

[33] Schaum N., et al. Single-cell transcriptomics of 20 mouse organs creates a Tabula Muris. Nature 2018, 562, 367–372. doi: 10.1038/s41586-018-0590-4 30283141 PMC6642641

[34] Hubert L. and Arabie P. Comparing partitions. J. Classif. 1985, 2, 193–218.

[35] Strehl A. and Ghosh J. Cluster ensembles—a knowledge reuse framework for combining multiple partitions. J. Mach. Learn. Res. 2002, 3, 583–617.

[36] Rousseeuw P.J. A graphical aid to the interpretation and validation of cluster analysis. J. Comput. Appl. Math. 1987, 20, 53–65.

[37] Davies D. L. and Bouldin D. W. A Cluster Separation Measure. IEEE Trans. Pattern Anal. Mach. Intell., 1979, PAMI-1, 224,227. 21868852

[38] Coifman R., et al. Geometric diffusions as a tool for harmonic analysis and structure definition of data: Diffusion maps. Proc. Natl. Acad. Sci. U.S.A 2005, 102, 7426–7431. doi: 10.1073/pnas.0500334102 15899970 PMC1140422

[39] Haghverdi L., et al. Diffusion pseudotime robustly reconstructs lineage branching. Nat. Methods 2016, 13, 845–848. doi: 10.1038/nmeth.3971 27571553

[40] McInnes L., et al. UMAP: Uniform Manifold Approximation and Projection for Dimension Reduction. arXiv 2018, arXiv:1802.03426v3. Available from: https://arxiv.org/abs/1802.03426

[41] Gambardella G., et al. A single-cell analysis of breast cancer cell lines to study tumour heterogeneity and drug response. Nat. Commun. 2022, 13, 1714. doi: 10.1038/s41467-022-29358-6 35361816 PMC8971486

[42] Barlund M., et al. Cloning of BCAS3 (17q23) and BCAS4 (20q13) genes that undergo amplification, overexpression, and fusion in breast cancer. Genes Chromosomes Cancer 2002, 35, 311–317. doi: 10.1002/gcc.10121 12378525

[43] Zhou Z., et al. BCAS3 exhibits oncogenic properties by promoting CRL4A-mediated ubiquitination of p53 in breast cancer. Cell Prolif. 2021, 54, e13088. doi: 10.1111/cpr.13088 34240781 PMC8349660

[44] Capes-Davis A., et al. Check your cultures! A list of cross-contaminated or misidentified cell lines. Int. J. Cancer 2010, 127, 1–8. doi: 10.1002/ijc.25242 20143388

[45] Ben-David U., et al. Genetic and transcriptional evolution alters cancer cell line drug response. Nature 2018, 560, 325–330. doi: 10.1038/s41586-018-0409-3 30089904 PMC6522222

[46] Bu D., et al. KOBAS-i: intelligent prioritization and exploratory visualization of biological functions for gene enrichment analysis. Nucleic Acids Res. 2021, 49, W317–W325. doi: 10.1093/nar/gkab447 34086934 PMC8265193

[47] Han X., et al. Mapping the Mouse Cell Atlas by Microwell-Seq. Cell 2018, 172, 1091–1107 e1017. doi: 10.1016/j.cell.2018.02.001 29474909

[48] Han X., et al. Construction of a human cell landscape at single-cell level. Nature 2020, 581, 303–309. doi: 10.1038/s41586-020-2157-4 32214235

[49] Jin S., et al. Inference and analysis of cell-cell communication using CellChat. Nat. Commun. 2021, 12, 1088. doi: 10.1038/s41467-021-21246-9 33597522 PMC7889871

[50] Seninge L., et al. VEGA is an interpretable generative model for inferring biological network activity in single-cell transcriptomics. Nat. Commun. 2021, 12, 5684. doi: 10.1038/s41467-021-26017-0 34584103 PMC8478947

