# Supplementary figures and images for: Attention-based deep clustering method for scRNA-seq cell type identification
Source: PLoS Comput Biol. 2023 Nov 10;19(11):e1011641. doi: 10.1371/journal.pcbi.1011641 (PMC10703402; doi:10.1371/journal.pcbi.1011641)

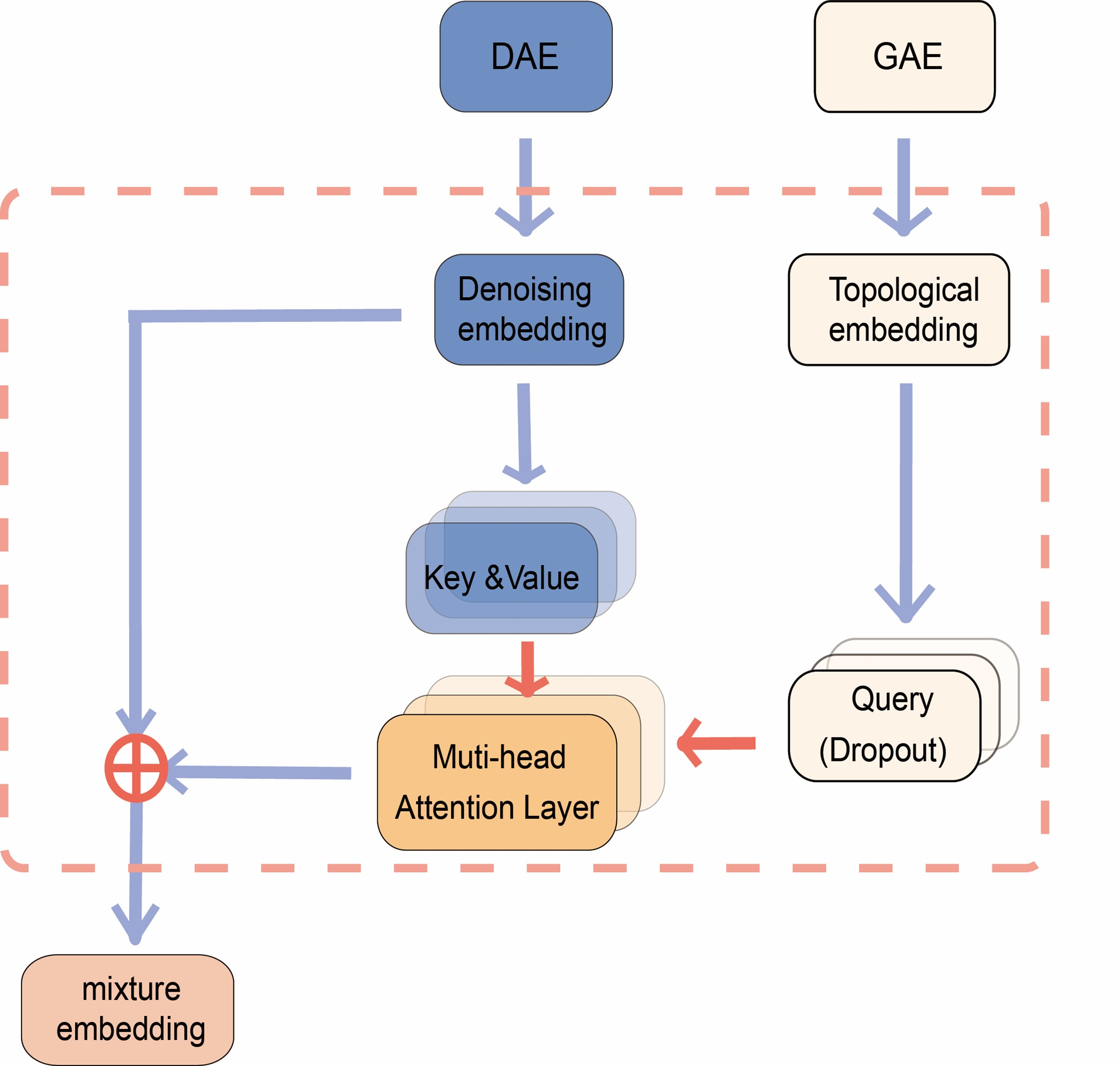

Supplement: S1 Fig — Different embeddings from DAE and GAE are combined by a multi-head attention layer and residual connection is adopted to promote the training efficiency of DAE module. (TIF) [file pcbi.1011641.s001.tif]

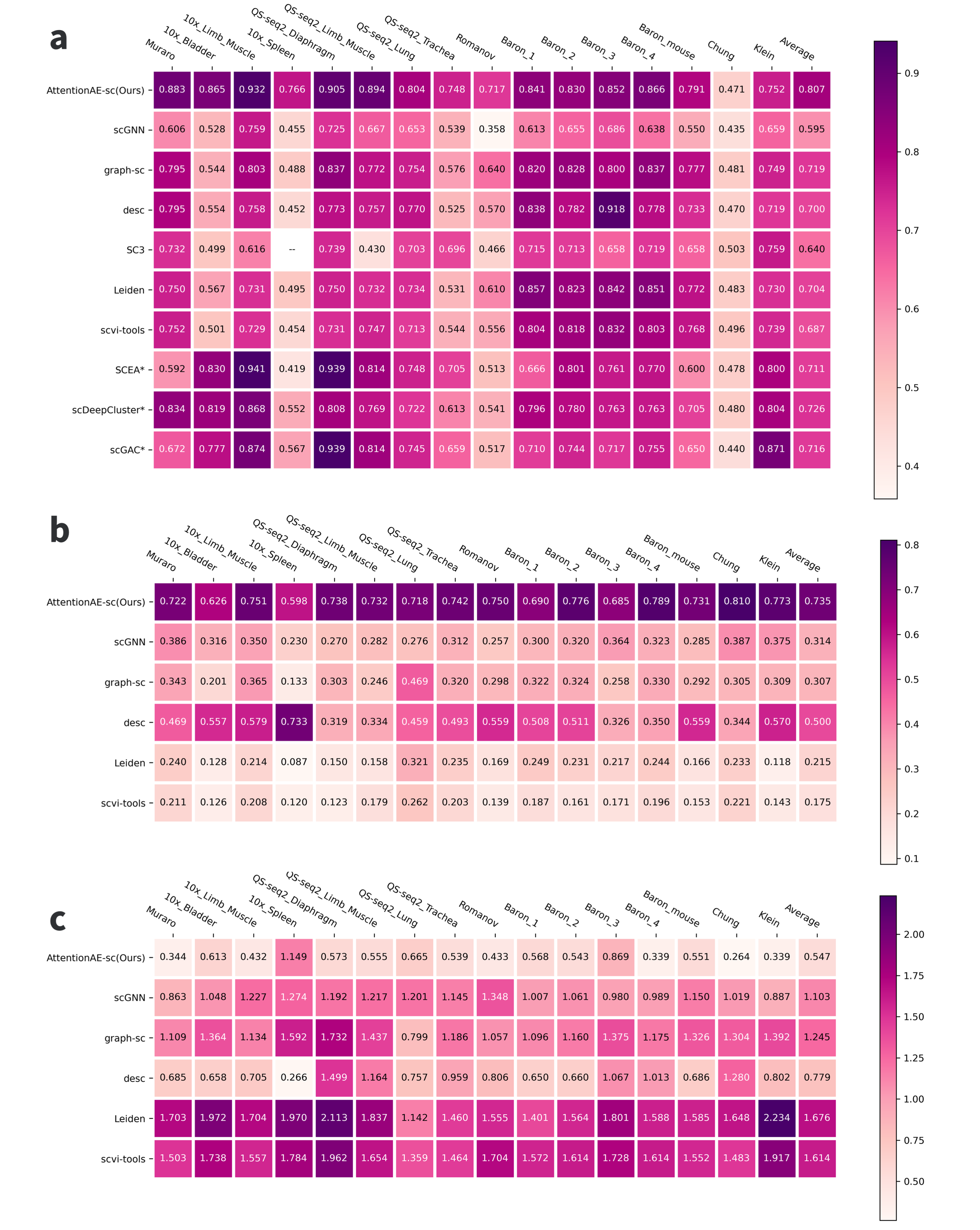

Supplement: S2 Fig — a. NMI scores of AttentionAE-sc and 9 baseline methods. b. Silhouette scores of 6 community detection-based methods (including AttentionAE-sc). c. Davies-Bouldin score of 6 community detection-based methods (including AttentionAE-sc). Arithmetic mean is taken as results of each dataset after running each method five times under different random seeds. Methods that need to specify the number of clusters are marked with an asterisk (*). (TIF) [file pcbi.1011641.s002.tif]

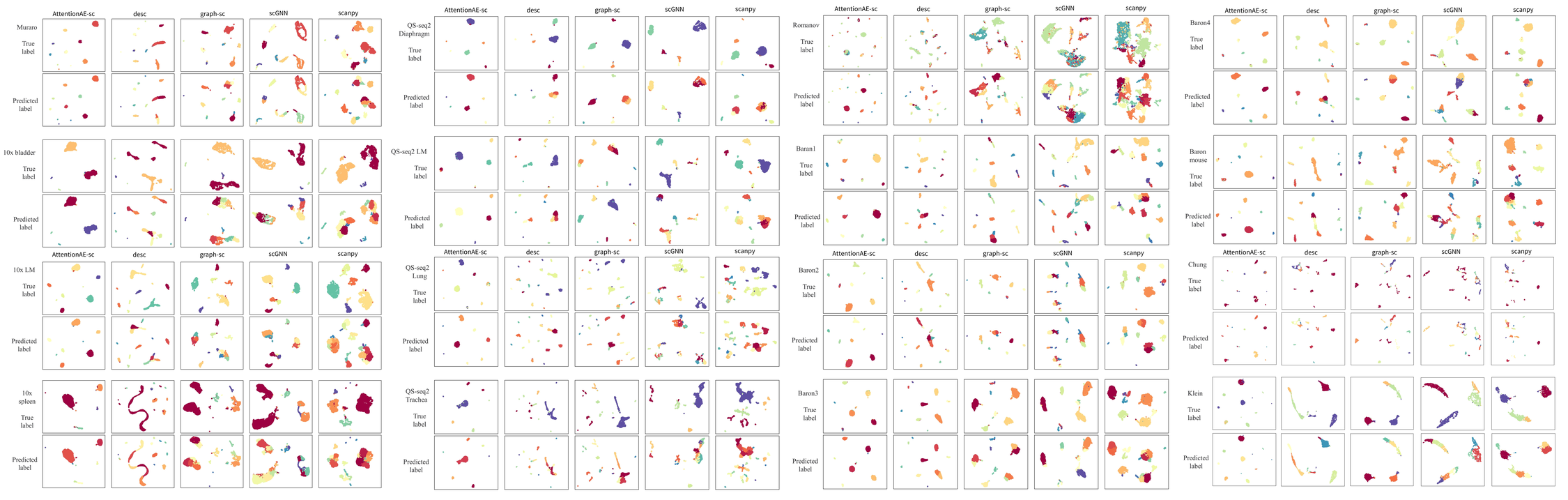

Supplement: S3 Fig — The 5 subfigures in each row are the cell projections obtained from the 2-dimentional UMAP features, which are calculated from the cell representation obtained by the different methods. Each dataset consists of 2 rows of subfigures that adopt the ground truth or predicted labels by different method respectively (different color clusters in the subfigure). In summary, AttentionAE-sc obtained the larger distinction between each group and the degree of internal tightness and the cell annotation is closer to the ground truth. (TIF) [file pcbi.1011641.s003.tif]

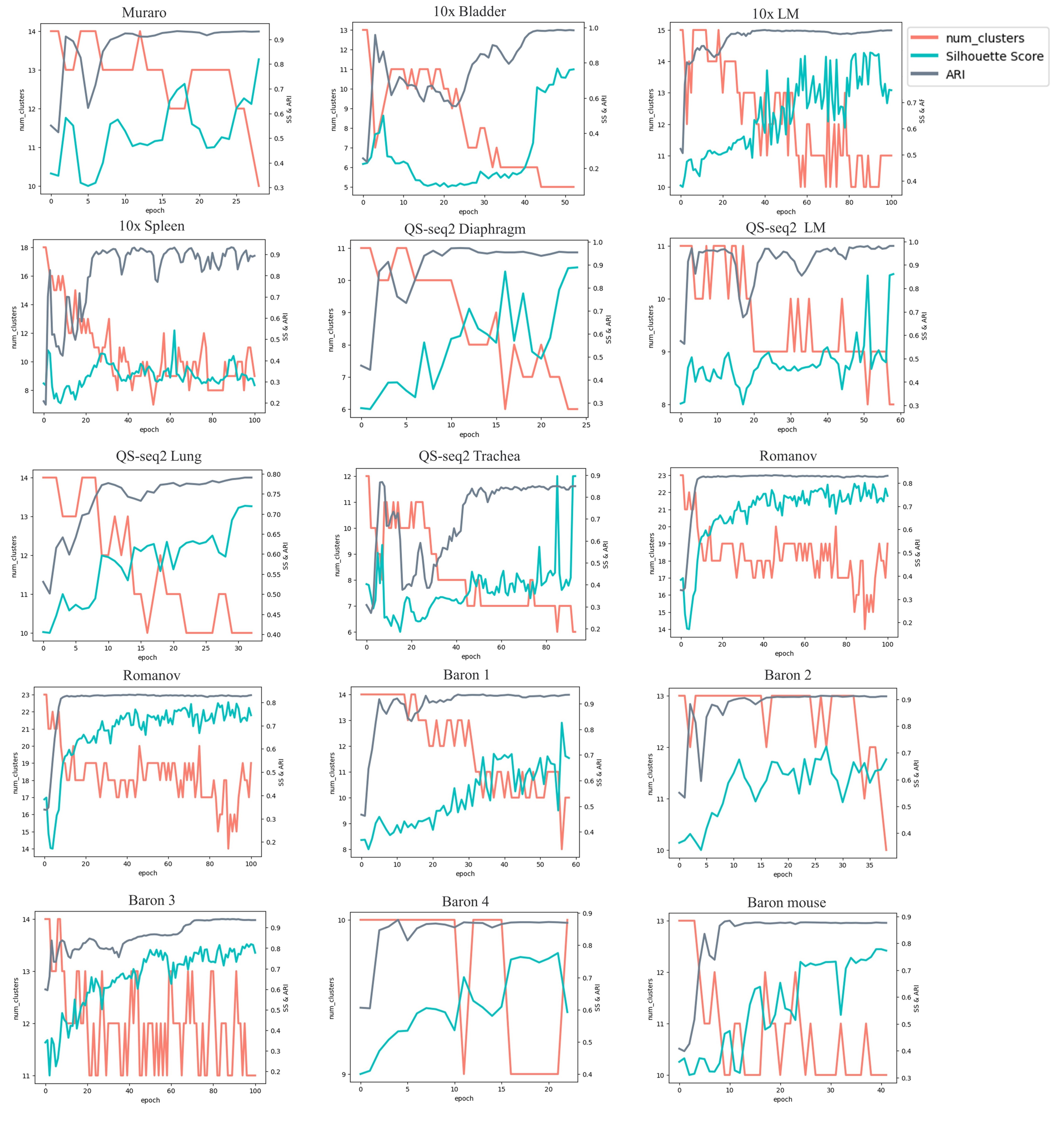

Supplement: S4 Fig — (TIF) [file pcbi.1011641.s004.tif]

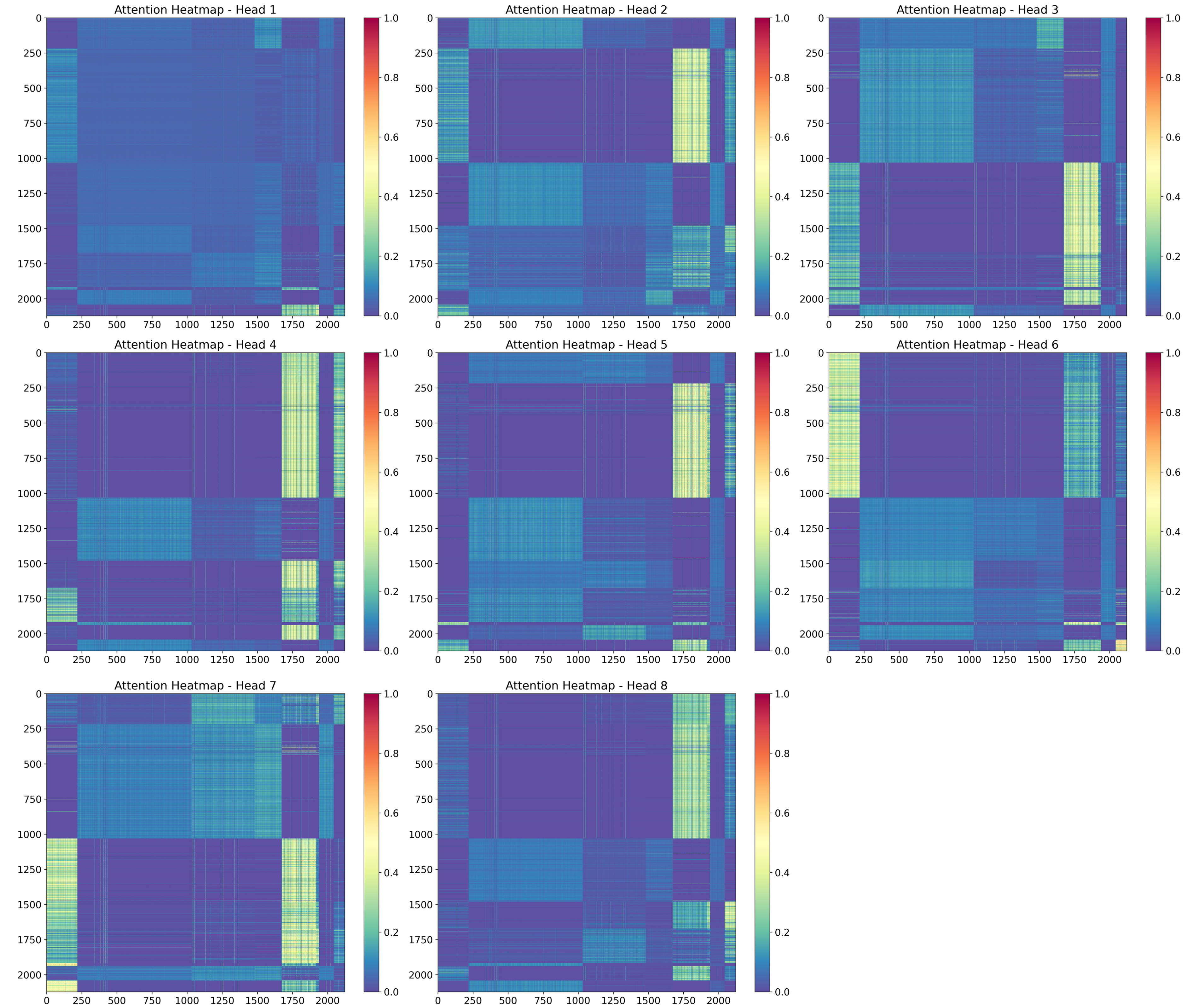

Supplement: S5 Fig — (TIF) [file pcbi.1011641.s005.tif]

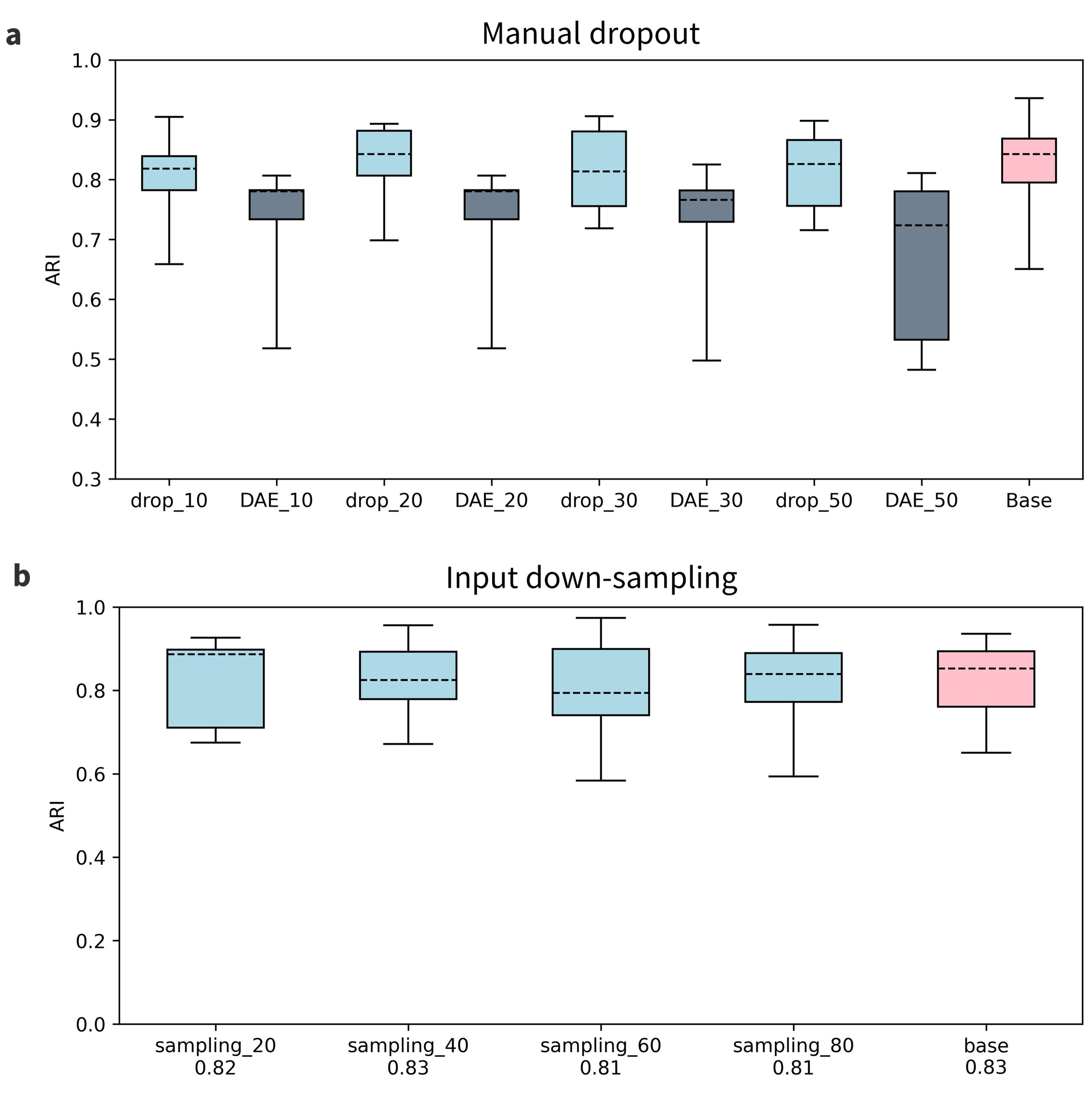

Supplement: S6 Fig — a. The ARI score of clustering results with random manual dropout rate of gene expression values set as 10%, 20%, 30% and 50% in the scRNA-seq datasets. All experiments run 5 times with different random seeds. Base (the pink box) is the performance of AttentionAE-sc without manual dropout. Compared AttentionAE-sc (drop 10 to drop 50, the blue box) with ordinary DAE (DAE 10 to DAE 50, the grey box), AttentionAE-sc is less influenced. b. The ARI score of clustering results with random and stratified input down-sampling rate of cells set as 20%, 40%, 60% and 80% respectively in the scRNA-seq datasets. All experiments run 5 times with different random seeds. Base (the pink box) is the performance of AttentionAE-sc with the whole scRNA-seq datasets as input. The average score is displayed below the x label on each box. (TIF) [file pcbi.1011641.s006.tif]

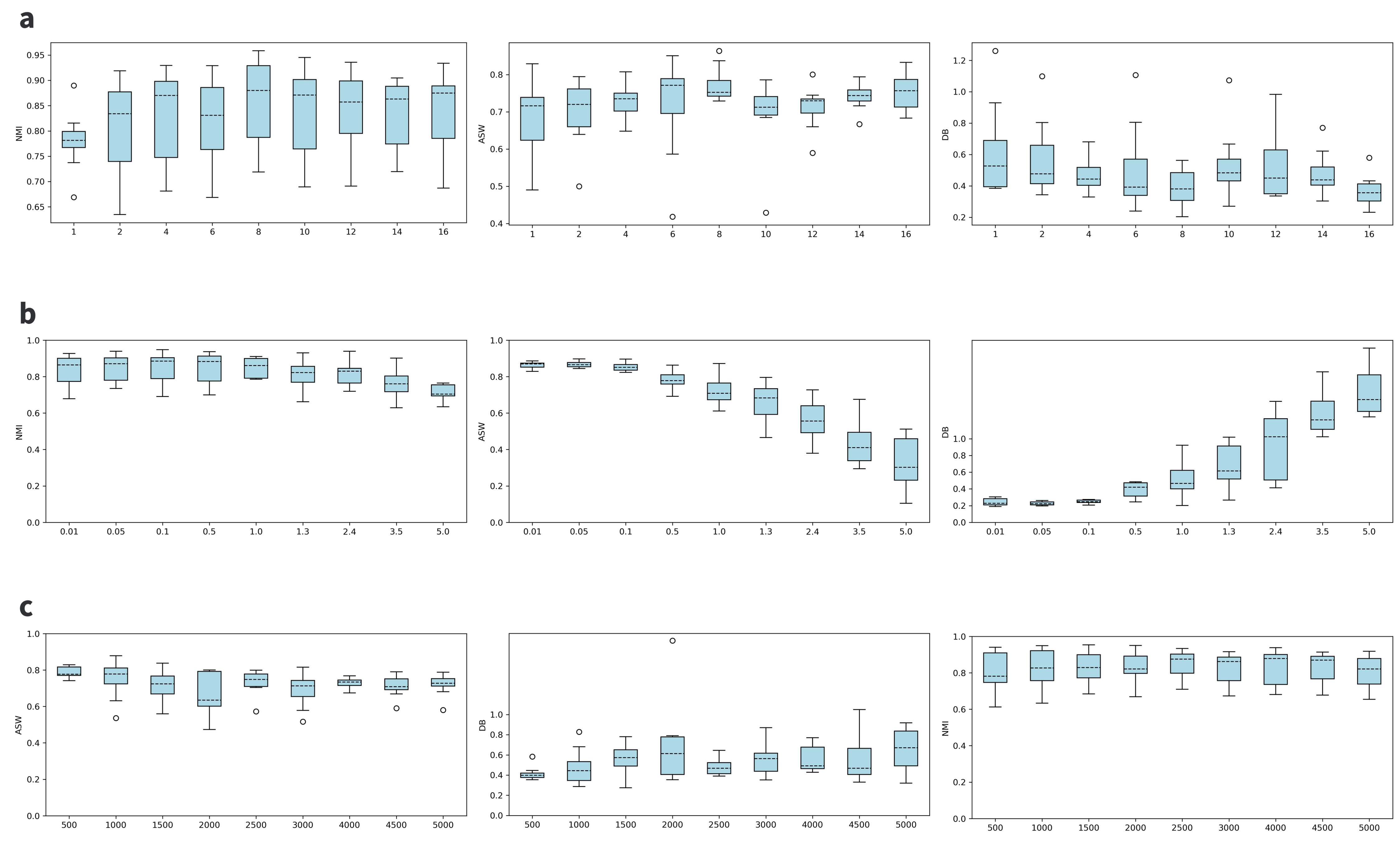

Supplement: S7 Fig — Three hyperparameters significantly influence the clustering performance of AttentionAE-sc: the number of attention heads in the information fusion blocks (a), the resolution parameters in the Leiden algorithm (b), and the number of highly variable genes (c). (TIF) [file pcbi.1011641.s007.tif]

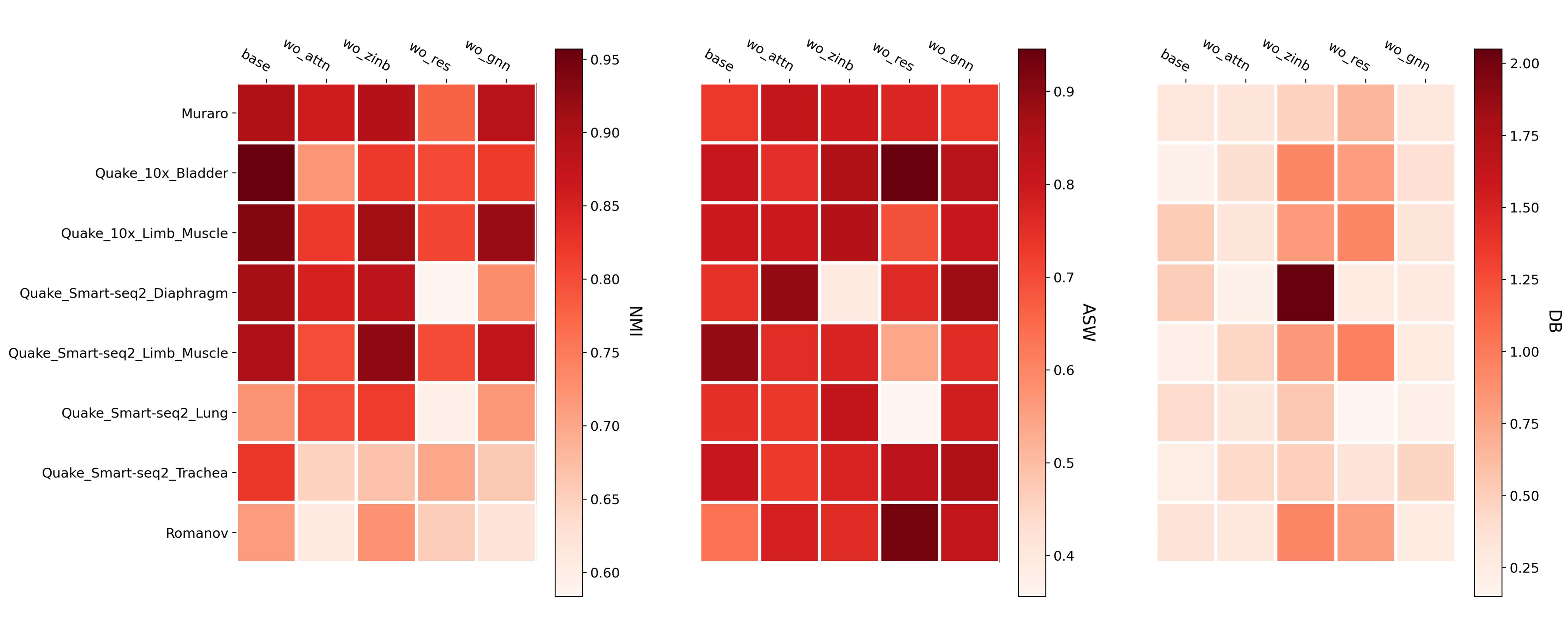

Supplement: S8 Fig — NMI, silhouette score (ASW) and Davies-Bouldin score (DB) are used as metrics. Four conditions were tested, including the absence of the information fusion block (wo attn), the absence of the ZINB loss function (wo zinb), the absence of residual connections (wo res), and the absence of GAE (wo gnn). (TIF) [file pcbi.1011641.s008.tif]

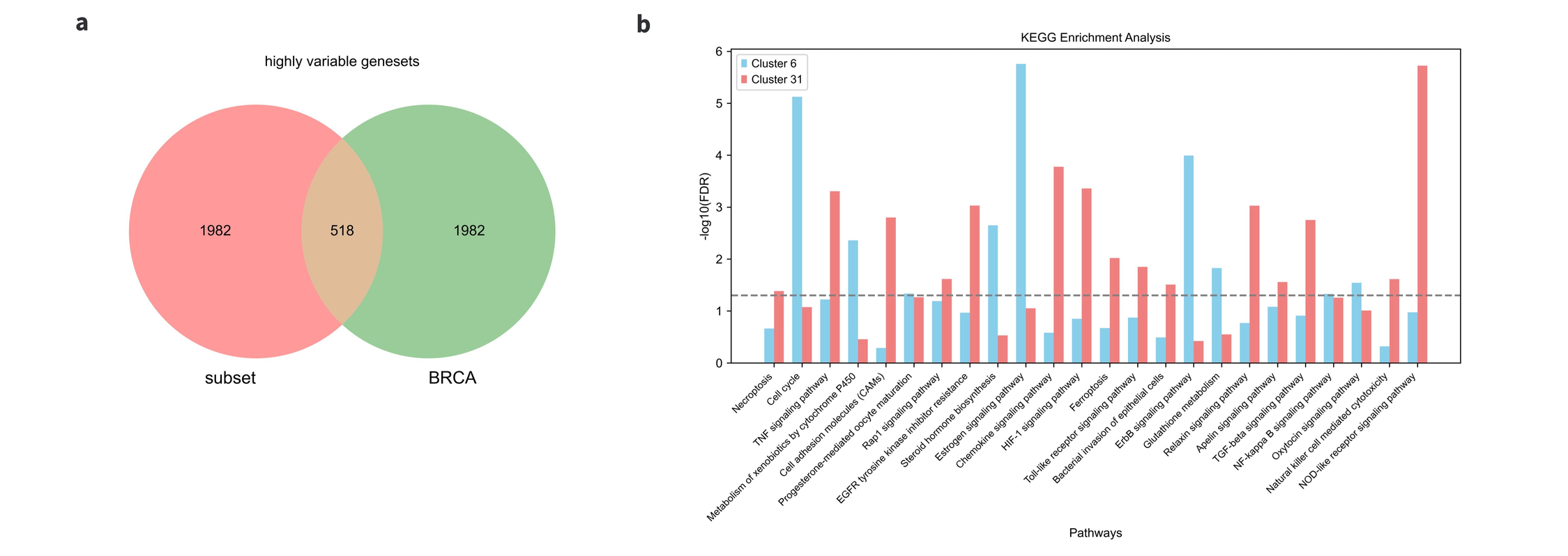

Supplement: S9 Fig — a. Venn diagram comparing the difference of highly variable genes after the preprocessing process between original clustering stage and sub-clustering stage. b. The results of KEGG enrichment analysis in the cluster 6 and cluster 31 based on the acquired DEGs in the S2 Table. In the histogram, the dotted line represents a threshold that the corrected p-value is 0.05 and the higher scores indicates significant enrichment. (TIF) [file pcbi.1011641.s009.tif]

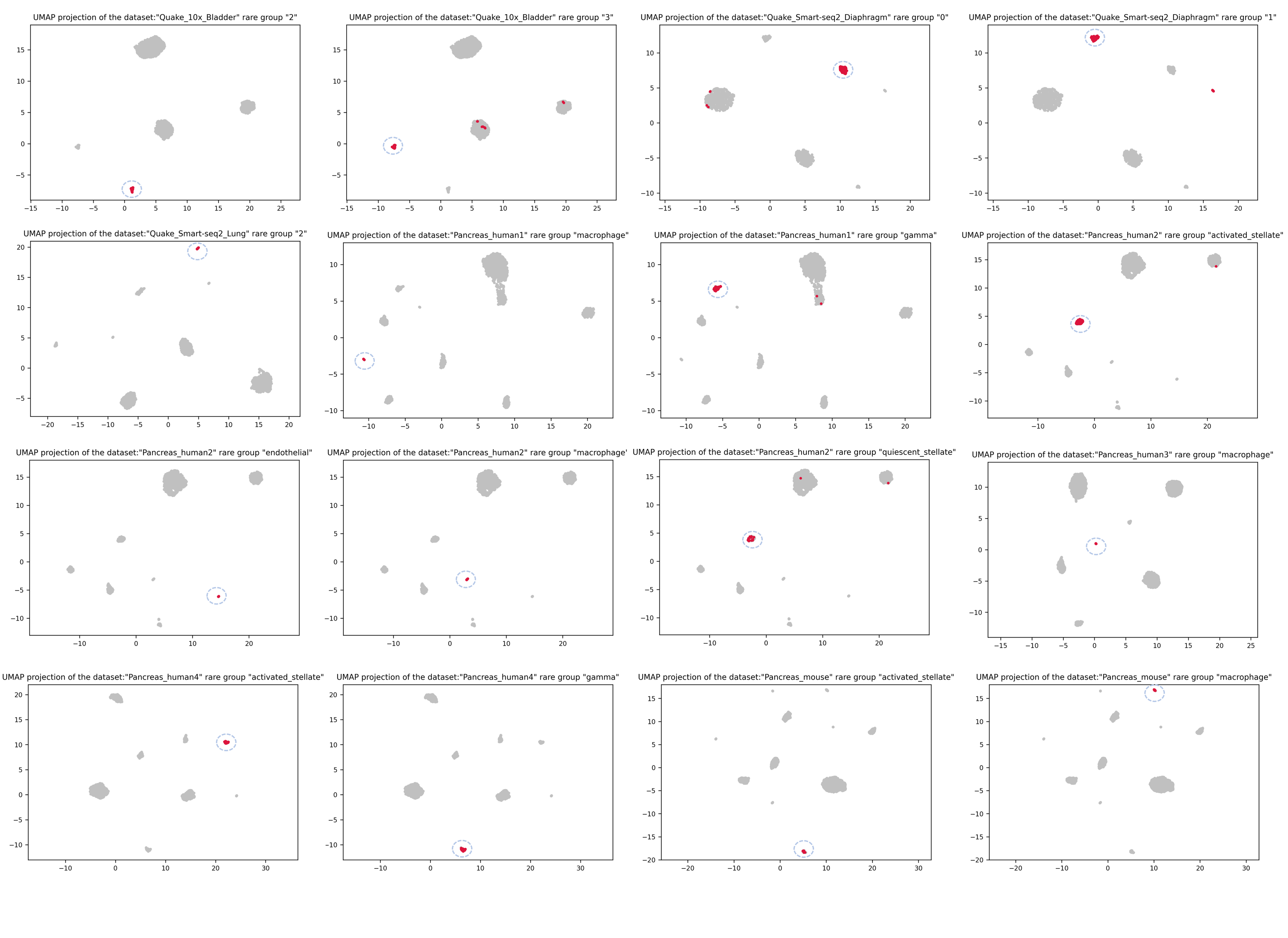

Supplement: S10 Fig — To assess the capacity for recognizing rare cell types, we opted to visualize cell types with a limited number of cells in the datasets. The results demonstrate that AttentionAE-sc possesses the capability to predict clusters with a small number of cells and distinguish rare cell types. (TIF) [file pcbi.1011641.s010.tif]
