# Supplementary material for: Attention-based deep clustering method for scRNA-seq cell type identification
Source: PLoS Comput Biol. 2023 Nov 10;19(11):e1011641. doi: 10.1371/journal.pcbi.1011641 (PMC10703402; doi:10.1371/journal.pcbi.1011641)
Supplement: S1 Table — Overview contains code source and operational environment for experiment in the real scRNA-seq datasets. (DOCX) [file pcbi.1011641.s011.docx]

**S1 Table.** General information of 9 state-of-art baseline methods. Overview contains code source and operational environment for experiment in the real scRNA-seq datasets.

| Methods | Github link | Experiment environment |
| --- | --- | --- |
| scGNN | https://github.com/juexinwang/scGNN | python=3.9.12  pytorch=1.12.1  scanpy=1.8.2 |
| graph-sc | https://github.com/ciortanmadalina/graph-sc | python=3.9.12  pytorch=1.12.1  scanpy=1.8.2 |
| desc | https://github.com/eleozzr/desc | python=3.8.17  tensorflow=2.2.0  keras=2.8.0  scanpy=1.8.2 |
| Leiden  (implemented by scanpy) | https://github.com/theislab/scanpy | python=3.9.12  scanpy=1.8.2  leidenalg=0.9.0 |
| scDeepCluster | https://github.com/ttgump/scDeepCluster | python=3.8.17  pytorch=1.11.0  scanpy=1.9.3  cudatoolkit=11.3.1 |
| Scvi-tools | https://github.com/scverse/scvi-tools | python=3.8.16  pytorch=2.0.1  torchmetrics=0.5.0  scvi-tools=0.14.6 |
| scGAC | https://github.com/Joye9285/scGAC | python 3.6.4  tensorflow=1.12.0  keras=2.1.0 |
| SCEA | https://github.com/SAkbari93/SCEA/tree/master | python 3.6.4  tensorflow=1.12.0  keras=2.1.0 |
| SC3 | https://bioconductor.org/packages/release/bioc/html/SC3.html | R=4.2.2  SC3=1.26.2  SingleCellExperiment=1.20.1 |
