# Supplementary material for: Attention-based deep clustering method for scRNA-seq cell type identification
Source: PLoS Comput Biol. 2023 Nov 10;19(11):e1011641. doi: 10.1371/journal.pcbi.1011641 (PMC10703402; doi:10.1371/journal.pcbi.1011641)
Supplement: S2 Table — For each clustering, the threshold for considering the presence of differential expression is 0.05 (P-values in Wilcoxon test). (DOCX) [file pcbi.1011641.s012.docx]

**S2 Table.** Differentially expressed genes between cluster 6 and cluster 31 in the breast cancer single-cell atlas experiment. For each clustering, the threshold for considering the presence of differential expression is 0.05 (P-values in Wilcoxon test)

| Cluster 6 | Cluster 31 |
| --- | --- |
| 'PTHLH', 'SERPINA1', 'SLPI', 'KPNA2', 'KLK10', 'PTTG1', 'MGST2', 'SERPINB3', 'KRT17', 'RRM2', 'C12orf75', 'KCNK6', 'AURKA', 'DSC3', 'SOX9', 'CDK1', 'H1-3', 'ITGB6', 'TSPAN1', 'IRX2', 'HBEGF', 'KIF16B', 'CDKN1A', 'PSMD6', 'RNF144B', 'GSTP1', 'PSMB3', 'PLAT', 'CXCL14', 'EREG', 'FAM110A', 'KIAA0040', 'AKR1B10', 'TINAGL1', 'TIMP3', 'MIR205HG', 'ALDH1A3', 'PGF', 'TPRG1', 'TNFSF10', 'S100A2', 'NEK2', 'GPRC5A', 'LAMA3', 'AKR1C3', 'CCND1', 'IGFL2-AS1', 'ARL6IP1', 'HSD17B2', 'CLDN1', 'KIF14', 'SAT1', 'HSPB1', 'FAM111B', 'CAV1', 'PSMD3', 'S100A6', 'KLK5', 'WFDC2', 'ID1', 'MED24', 'PFDN4', 'TMEM40', 'FYB1', 'BTG2', 'NMU', 'CCNB1', 'SERPINB5', 'FABP5', 'CDC20', 'MT1E', 'RHOD', 'RGS16', 'FAM83A', 'TACSTD2', 'THBD', 'TMSB4X', 'LY6D', 'FBXO2', 'LCN2', 'EPPK1', 'LACTB2', 'S100A7', 'MAL2', 'SEC61G', 'SYNE2', 'SERPINB13', 'CLCA2', 'COBLL1', 'LAMC2', 'MRPL21', 'IL18', 'IFI16', 'MT2A', 'SPRR1B', 'AKR1C1', 'IL20RB', 'NUF2', 'VSNL1', 'LYN', 'NRG1', 'MT1L', 'KRT6A', 'TOP2A', 'HSPA1A', 'SGK1', 'CSRP2', 'AQP3', 'KRT6B', 'ARL4C', 'S100A9', 'STEAP1', 'AREG', 'HMMR', 'IL36G', 'CENPF', 'CISD3', 'CDKN3', 'KLRK1-AS1', 'FOS', 'HMGA2', 'H4C3', 'SCD', 'SAA2', 'MKI67', 'CKS2', 'PMAIP1', 'SNCG', 'ATF3', 'COL17A1', 'IL1B', 'TRIP13', 'NEFL', 'SFRP1', 'UBE2C', 'ASPM', 'GRB7', 'PTPRZ1', 'KRT14', 'CRABP2', 'UPK1B', 'CSTB', 'EGFR', 'FGFBP1', 'NDRG1', 'NUSAP1', 'JAG1', 'DSG3', 'CENPE', 'PLAU', 'BCL11A', 'ITGA2', 'CYSRT1', 'PHLDA1', 'FAM3C', 'CA2', 'H1-4', 'DUSP6', 'KRT16', 'MXD1', 'KLF6', 'SERPINB4', 'FST', 'CTSC', 'MT1X', 'SFN', 'GAL', 'AKR1C2', 'DST', 'PI3', 'NUPR1', 'DSP', 'CSTA', 'KRT23', 'GADD45A', 'IGFL1', 'LAMB3', 'INPP4B', 'S100A8', 'KRT15', 'COL12A1', 'FEZ1', 'SLC9A3R1', 'SAA1', 'TPX2', 'ITGB4', 'FERMT1', 'TM4SF1', 'AHNAK2', 'KRT5’, 'HES2' | 'FBN1', 'VEGFA', 'LINC03033', 'SULF2', 'PLXDC2', 'CTSL', 'ZFP36L1', 'MYLK', 'CCDC80', 'TIMP2', 'CLMP', 'PLEKHA4', 'ITM2C', 'COL6A1', 'SDC2', 'H2AC6', 'RHOBTB3', 'XIST', 'AKR1B1', 'PTGES', 'GAS6', 'H2AC25', 'FAP', 'MDK', 'TGFBI', 'SCG5', 'NEXN', 'DDIT4', 'SPOCK1', 'MAP1B', 'IL7R', 'FBXO32', 'BICC1', 'IFI6', 'COL6A2', 'MME', 'CPVL', 'CCBE1', 'HOXA9', 'VMP1', 'PAPPA', 'TENT5A', 'MLPH', 'ITGA5', 'WFDC21P', 'IGFBP4', 'TIMP1', 'DLC1', 'SLC4A7', 'CXCL3', 'CRIM1', 'GADD45B', 'CCN1', 'SOX4', 'LOX', 'ADAMTS6', 'CXCL8', 'C1R', 'VIM', 'CD70', 'MMP14', 'PDPN', 'CTSD', 'IGFBP7', 'PLAC8', 'MFGE8', 'SMIM14', 'MSC', 'ID3', 'DUSP4', 'KYNU', 'RND3', 'IGFBP3', 'CD99L2', 'DUSP1', 'DAB2', 'MAGED2', 'CA9', 'CDA', 'MAFB', 'LINC02015', 'HLA-C', 'CTSB', 'HEG1', 'TXNIP', 'CDH2', 'SMOX', 'EVI2A', 'TMEM45A', 'BMERB1', 'SQSTM1', 'GBP1', 'VCAN', 'RPL23', 'SPEF2', 'GDF15', 'DPYSL2', 'FTH1', 'NREP', 'CDC42EP3', 'TNC', 'MSRB3', 'SPARC', 'TUBA1A', 'CXCL1', 'DPP4', 'COL5A1', 'ABCC2', 'PTPRE', 'MYL9', 'S100A4', 'CCN2', 'TPM2', 'CST3', 'STAT1', 'TGFB2', 'TPM1', 'PF4', 'CYP1B1', 'RAMP1', 'ZEB1', 'ABCA1', 'PLPP4', 'EFEMP1', 'SERPINE1', 'CCN5', 'TFPI', 'RPS24', 'GNAI1', 'NRP2', 'PXDN', 'G0S2', 'ANGPT1', 'SMARCA1', 'TREM1', 'FGF2', 'SBSN', 'CCNG2', 'TFPI2', 'STC1', 'HACD1', 'CDH13', 'STC2', 'TRAM2', 'SERPINE2', 'KRT8', 'LAMA4', 'LOXL2', 'RPL22L1', 'GBP2', 'COL4A1', 'PLIN2', 'FN1', 'ADM', 'LMO4', 'PXK', 'FTL', 'NNMT', 'SH3BGRL', 'MCAM', 'AKAP9', 'SERPINH1', 'TRPS1', 'MSC-AS1', 'PIK3R1', 'CLIC3', 'MAP2', 'SELENOM', 'NUCB2', 'HLA-B', 'COL1A1', 'COL4A2', 'HLA-A', 'WNT5A', 'TAOK1', 'APOC1', 'ANPEP', 'FSTL1', 'FERMT2', 'LTBP1', 'ID2', 'CPA4', 'SRGN', 'CLU', 'OLFML2B', 'RPL19', 'AMIGO2', 'CHI3L1', 'RBMS3', 'HOXA5', 'C14orf132', 'CALD1', 'GOLM1', 'SLC39A14', 'NFKBIZ', 'C1S', 'IGFBP6', 'ABCC3', 'CXCL2', 'IL6', 'TLE4', 'PRSS23', 'BGN' |
