# Supplementary material for: Attention-based deep clustering method for scRNA-seq cell type identification
Source: PLoS Comput Biol. 2023 Nov 10;19(11):e1011641. doi: 10.1371/journal.pcbi.1011641 (PMC10703402; doi:10.1371/journal.pcbi.1011641)
Supplement: S3 Table — Compare with another GNN-based methods in the 14 real scRNA-seq datasets. Nvidia RTX 3070Ti (8 GB RAM) was used in the gpu test. (DOCX) [file pcbi.1011641.s013.docx]

**S3 Table.** Elapsed time (sec) in the train process. Compare with another GNN-based methods in the 14 real scRNA-seq datasets. Nvidia RTX 3070Ti (8 GB RAM) was used in the gpu test.

| Dataset | Ours | | scGAC | scGNN |
| --- | --- | --- | --- | --- |
|  | cpu | gpu |  |  |
| Muraro | 344.888 | 100.993 | 161.155 | 166.9297 |
| Quake_10x_Bladder | 568.3493 | 133.116 | 229.4558 | 194.6873 |
| Quake_10x_Limb_Muscle | 828.4453 | 172.9954 | 537.4308 | 271.4626 |
| Quake_10x_Spleen | 1250.844 | 268.3782 | 3617.655 | 820.9404 |
| Quake_Smart-seq2_Diaphragm | 122.2737 | 37.7236 | 42.50784 | 80.99025 |
| Quake_Smart-seq2_Limb_Muscle | 138.1951 | 57.8016 | 59.58744 | 90.41449 |
| Quake_Smart-seq2_Lung | 255.0625 | 73.6566 | 121.0019 | 131.9925 |
| Quake_Smart-seq2_Trachea | 247.3509 | 83.1443 | 88.3029 | 102.3073 |
| Romanov | 659.5955 | 163.8671 | 310.3852 | 209.6446 |
| Pancreas_human1 | 394.3007 | 113.5227 | 155.645 | 136.3506 |
| Pancreas_human2 | 344.2684 | 77.7262 | 136.1335 | 127.7251 |
| Pancreas_human3 | 910.4006 | 224.233 | 475.407 | 263.8218 |
| Pancreas_human4 | 177.5092 | 53.9511 | 100.6044 | 101.9777 |
| Pancreas_mouse | 269.5487 | 75.1074 | 161.5822 | 137.0036 |
